# Electrocatalytic Hydrogen Oxidation by Defect‐Enriched CuW Nanoalloy Featuring Hydrogen Spillover Effect

**DOI:** 10.1002/advs.202503710

**Published:** 2025-06-20

**Authors:** Bingyan Xiong, Lisong Chen, Jianlin Shi

**Affiliations:** ^1^ School of Medicine Tongji University Shanghai 200331 P. R. China; ^2^ Shanghai Key Laboratory of Green Chemistry and Chemical Processes School of Chemistry and Molecular Engineering East China Normal University Shanghai 200062 P. R. China; ^3^ Shanghai Institute of Ceramics Chinese Academy of Sciences Shanghai 200050 P. R. China

**Keywords:** CuW nanoalloy, defects, electro‐deposition, hydrogen oxidation reaction, hydrogen spillover effect

## Abstract

The development of efficient alkali‐resistant non‐noble metal electrocatalysts for hydrogen (H_2_) oxidation reaction (HOR) is crucial for advancing the hydrogen economy. Recently, the hydrogen spillover effect has been proven to be one of the vital means to enhance the activity of the HOR catalyst. In this report, a facile and controllable electrodeposition strategy is proposed for synthesizing a tungsten (W)‐doped copper (Cu) nanoalloy/WO_3_ electrocatalyst with abundant metal‐metal heterointerfaces to promote hydrogen spillover, i. e., the migration of ^*^H from W/WO_3_ sites to Cu substrates. CuW grown in situ on carbon paper (CP) demonstrates exceptional HOR performance (1.86 mA cm^−2^ at 0.05 V vs. RHE) and remarkable stability in alkaline media. XAFS and density functional theory (DFT) calculations confirm that alloying W atoms into the Cu lattice induces electronic modulation, significantly boosting the adsorption, decomposition, and oxidation of H_2_. The migration of ^*^H from W/WO_3_ sites to Cu sites via the hydrogen spillover effect has been validated by *in*‐*situ* Fourier transform infrared (FT‐IR) and Raman spectra. This study may open up a new path for designing high‐efficiency HOR electrocatalysts from the perspective of appropriate hydrogen spillover channel design.

## Graphical abstract

The CuW nanoalloy is synthesized via electrodeposition, with the coexistence of Cu, W/WO_3_ enhancing hydrogen spillover and improving hydrogen oxidation. W/WO_3_ facilitates hydrogen adsorption, whereas Cu supports hydrogen spillover and oxidation catalysis. The combination of the two effectively overcomes the bottleneck of hydrogen spillover in traditional catalytic processes.

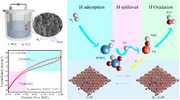

## 1 Introduction

H_2_ is regarded as the most promising energy carrier to replace fossil fuels owing to its high energy density, renewability, and environmental friendliness.^[^ ^1^ ^]^ The HOR, which plays a pivotal role in converting H₂ into usable energy, directly determines the efficiency of hydrogen utilization. In acidic environments, platinum‐group metals (PGMs) such as Pt remain indispensable as the primary active components in catalysts due to their exceptional corrosion resistance. In contrast, alkaline media offer the potential for non‐PGM electrocatalysts to drive HOR, as these conditions inherently enhance material stability and reduce costs for hydrogen energy applications.^[^ ^2^ ^]^ Despite these advantages, the sluggish reaction kinetics in alkaline environments result in significantly diminished HOR activity for most electrocatalysts, including even state‐of‐the‐art PGM‐based systems.^[^ ^3^ ^]^

H_2_ spillover is a catalytic phenomenon where active H species (^*^H), generated through the adsorption and decomposition of H_2_ on the surface of metal particles surfaces, migrate to the supporting substrate. This process enables the efficient transfer of excess ^*^H from metal nanoparticles, thereby maintaining optimal catalytic activity in hydrogen‐related reactions.^[^ ^4^, ^5^ ^]^ Capitalizing on this mechanism, researchers have developed various metal heterointerfaces through the fabrication of alloyed, metallic, or metal oxide‐supported electrocatalysts to enhance hydrogen spillover and subsequently improve the HOR performance.^[^ ^6^, ^7^, ^8^, ^9^, ^10^ ^]^ Researches have also revealed that introducing edge dislocations in single‐phase catalysts can effectively enhance the hydrogen spillover effect.^[^ ^11^ ^]^ Among non‐precious metal elements utilized in catalyst design, W stands out due to its exceptional electrical conductivity and complex chemical states with accessible d and f orbitals.^[^ ^12^, ^13^ ^]^ Furthermore, W demonstrates remarkable capabilities in promoting H_2_ dissociation, facilitating ^*^H adsorption, and enabling efficient ^*^H transfer from active sites to the substrate, thereby significantly contributing to the hydrogen spillover process. These unique properties have led to the development of various W‐based HOR electrocatalysts, including W‐Ni_3_N (≈ 1.80 mA cm^−2^ at 0.05 V vs. RHE), Co_3_W‐WNi_4_ (≈ 2.00 mA cm^−2^ at 0.05 V vs. RHE), and WNi alloy (≈ 2.20 mA cm^−2^ at 0.05 V vs. RHE).^[^ ^2^, ^14^, ^15^ ^]^ More importantly, tungsten nitride and its corresponding oxides have been effectively employed as catalytic supports to further enhance HOR activity.^[^ ^16^, ^17^ ^]^ Notably, tungsten (W)‐based catalysts demonstrate especially high HOR activity compared to some noble metal counterparts. For instance, while the Pd‐grown@Ni/NiO‐decorated fiber catalyst achieves a current density of ≈ 0.01 mA cm^−2^ at 0.05 V vs. RHE, and Pt‐Ce_4_ shows ≈ 2.20 mA cm^−2^ at 0.05 V vs. RHE, W‐based catalysts under identical conditions exhibit significantly higher current densities.^[^ ^18^, ^19^ ^]^ This performance advantage, coupled with the inherent cost‐effectiveness of W‐based materials, positions them as promising alternatives to noble metal catalysts for sustainable energy applications.

Previous studies have revealed significant differences in hydrogen spillover kinetics between different catalyst supports. Specifically, the chemical reaction kinetics of hydrogen spillover on catalysts supported by non‐reducible oxides are ≈ 10 orders of magnitude slower than those with reducible metal oxide supports.^[^ ^20^ ^]^ This substantial difference is attributed to the accumulation of unstable atomic H species and the absence of coherent proton‐electron transfer mechanisms in non‐reducible oxide systems.^[^ ^16^, ^20^ ^]^ In conclusion, reducible metal oxide offers strong capability in promoting hydrogen spillover through these coherent proton‐electron movements, however, their practical application is often limited by inherently low conductivity, which typically results in reduced catalytic reaction current densities and energy conversion efficiency.^[^ ^4^, ^21^ ^]^ To address these limitations and optimize catalytic performance, it is essential to design support materials that simultaneously facilitate efficient hydrogen spillover and maintain high electrical conductivity. This dual requirement necessitates the development of catalyst carriers that not only promote the hydrogen spillover process but also ensure rapid electron transfer from the catalytic active sites to the electrode. Such characteristics are crucial for maintaining high catalytic activity and achieving optimal reaction current densities.

Addressing these challenges, we constructed a heterostructured CuW/WO_3_ nanoalloy (≈ 5 nm) catalyst (CuW) using electrodeposition technology. Benefiting from the controllability of the electrodeposition method over catalyst morphology and the dynamic deposition process's inherent ability to spontaneously form grain boundaries and defects, which was absent in high‐temperature thermal treatments, hydrothermal methods and chemical reduction methods, the prepared catalyst particles were size‐controlled at 5 nm, while being rich in defects and heterointerfaces.^[^ ^22^, ^23^ ^]^ As catalyst support, Cu not only possessed intrinsic properties such as high abundance, low toxicity, and high electrical conductivity but more importantly, its surface was prone to weak oxidation, thereby endowing it with reducible characteristics that effectively facilitated hydrogen spillover processes.^[^ ^24^ ^]^ The test results show that W atoms are *in*‐*situ* doped into the Cu lattice in the catalyst and exists a CuW/WO₃ heterojunction structure. Mechanistic studies on the HOR catalytic process reveal that this unique catalyst configuration facilitates H_2_ adsorption on W‐related catalytic sites and enhances H species conversion on the Cu substrate. The presence of WO_3_ effectively promotes hydrogen spillover, thereby endowing the catalyst with exceptional HOR performance. These mechanistic insights not only validate our design strategy but also establish a novel paradigm for developing hydrogen spillover‐enhanced catalysts, particularly for HOR catalysis. This work represents a significant advancement in the rational design of high‐performance electrocatalysts through precise structural engineering and interface optimization.

## 2 Results and Discussion

### 2.1 Preparation and Characterization of CuW

**Figure 1:**
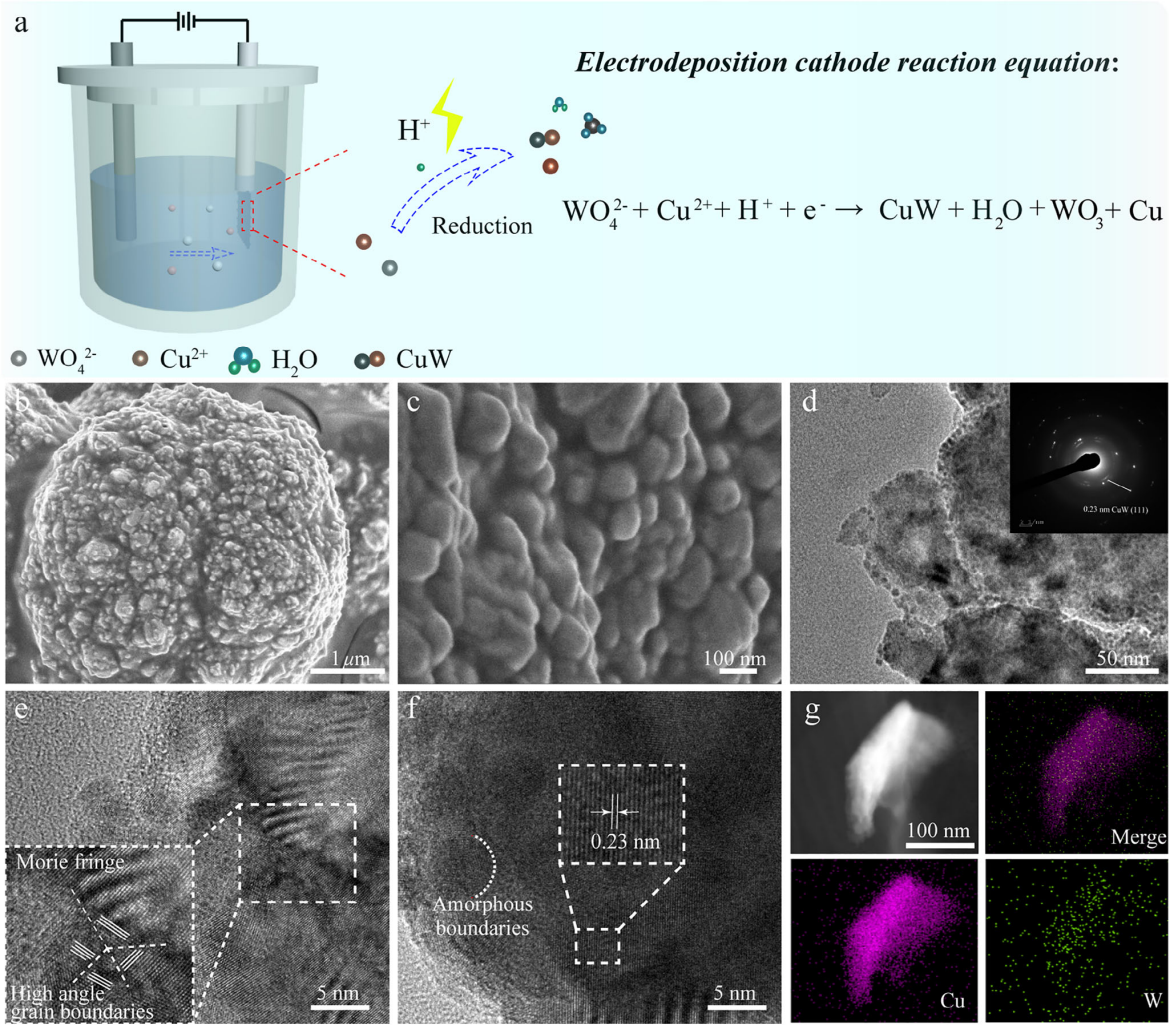
Schematic diagram of the synthetic process, structure, and characterizations of CuW. a) Schematic diagram of CuW synthetic process. b, c) SEM images of CuW particles. d) High‐definition TEM image of CuW, and the inset is the corresponding SAED pattern. e,f) Structure defects at high angle grain boundaries (e) and amorphous boundaries (f), Morie fringes (e, f) and the lattice planes of 0.23 nm in inter‐planar distance can be observed (f). g) STEM‐HAADF image, EDS elemental mapping images of CuW.

A precisely controlled nanoparticle structure is successfully fabricated through a facile and rapid electrodeposition protocol, as illustrated in **Figure** **1**a. The electrochemical system was configured with a carbon rod as the counter electrode, pre‐treated carbon paper as the working electrode, and an Ag/AgCl (saturated KCl) electrode as the reference. For a typical experimental process, the electrodeposition process was carried out in a N_2_‐saturated electrolyte containing Cu^2+^ and WO_4_^2−^ ions, maintained at 70 °C with constant stirring to ensure uniform ion distribution. The synthesis employed cyclic voltabsorptometry (CVA) with specific parameters: a potential window of −2.1 V to −0.6 V vs. Ag/AgCl, a scan rate of 100 mV s^−1^, and fixed at −2.1 and −0.6 V for 0.05 and 0.5 s, respectively, in each cycle. The total electro‐deposition time duration was 15 min. The detailed synthesis parameters were provided in the experimental section, offering comprehensive information on the material fabrication process.

In systematically investigate the optimal experimental conditions, a series of controlled experiments were conducted. For convenience, with sample nomenclature based on the molar ratios of Cu to W in the electrodeposition solution precursor. Initial observations reveal that when Na_2_WO_4_ served as the sole metal‐based reactant in the electrolyte, the reaction kinetics are notably sluggish, yielding only a sparse distribution of ≈ 20 nm nanoparticles on the CP substrate (Figure S1a,b, Supporting Information). However, upon introducing and progressively increasing the Cu ratio (*n*_Cu_:*n*_W_ = 3:14, 6:14, 12:14), a substantial *in*‐*situ* growth of nanoparticles is observed on the CP surface, with the most favorable particle morphology (≈ 70 nm) achieved at an optimal molar ratio of *n*_Cu_:*n*_W_ = 6:14 (Figure 1b,c; Figure S1c–f, Supporting Information). Contrastingly, when CuSO_4_ was employed as the exclusive metal‐based reactant, the resulting particles exhibit undesirable characteristics of large size and severe agglomeration (Figure S1g,h, Supporting Information). Further optimization studies demonstrate that under the ideal electrolyte ratio, the morphology of CuW nanoparticles show significant dependence on both experimental duration and temperature, with the most optimal morphological characteristics emerging at 70 °C with a 15‐min reaction time (Figures S2a–h and S3a–h, Supporting Information). This optimal condition is further substantiated by XRD analysis, which indicates that at a Cu to W molar ratio of 6:14 and a preparation temperature of 70 °C, W demonstrates superior doping efficiency in Cu compared to other tested ratios (3:14, 12:14) and temperatures (50, 60, 80 °C) (Figure S4 and Tables S1 and S2, Supporting Information).

The high‐resolution transmission electron microscopic (HRTEM) analysis, as depicted in Figure 1d, demonstrates the presence of numerous nanoparticles with an approximate diameter of 5 nm uniformly distributed on the surface of CuW. Besides, the selected‐area electron diffraction (SAED) image confirms a lattice spacing of 0.23 nm, corresponding to the (111) crystal plane of the CuW alloy. Furthermore, the existence of W atoms promotes the development of various defect structures, including amorphous boundaries, Moire fringes, and high‐angle grain boundaries, as evidenced in Figure 1e,f. Notably, such defect structures are absent in pure Cu and W samples, as shown in Figures S5 and S6 (Supporting Information). Detailed examination reveals that the nanoparticles exhibit a specific crystal face with an interplanar spacing of 0.23 nm (Figure 1f), which is notably larger than the d‐spacing of Cu (111) plane (0.21 nm) and W (110) plane (0.22 nm). This lattice expansion provides clear evidence of W atoms being successfully doped into the Cu lattice, resulting in lattice distortion and the formation of abundant heterointerfaces. The structural characteristics are further corroborated by high‐angle annular dark‐field scanning transmission electron microscopy (HAADF‐STEM) imaging and corresponding energy‐dispersive X‐ray spectroscopy (EDS) elemental mapping (Figure 1g), which confirm the homogeneous distribution of both Cu and W elements throughout the CuW nanoalloy material. To further confirm whether the amorphous regions of CuW contain oxygen elements, a Corrector for Lens Aberrations in a Scanning Transmission Electron Microscope (CLAC‐STEM) was conducted. The high‐resolution EDS mapping clearly reveals that oxygen is distributed not only in amorphous regions, but across the entire material (Figure S7, Supporting Information). Additionally, the CuW crystallites embedded within Cu nanocrystals, along with partial WO_3_ residues is confirmed by CLAC‐STEM (Figure S8, Supporting Information). This observation validates the existence of CuW while indicating limited W incorporation into the lattice and incomplete reduction of WO_3_. Besides, comparative XPS analyses before and after argon plasma etching unambiguously verify the existence of WO_3_ both on the surface and in deeper subsurface regions, with significantly reduced WO_3_ concentration in the interior (Figure S9, Supporting Information).

**Figure 2:**
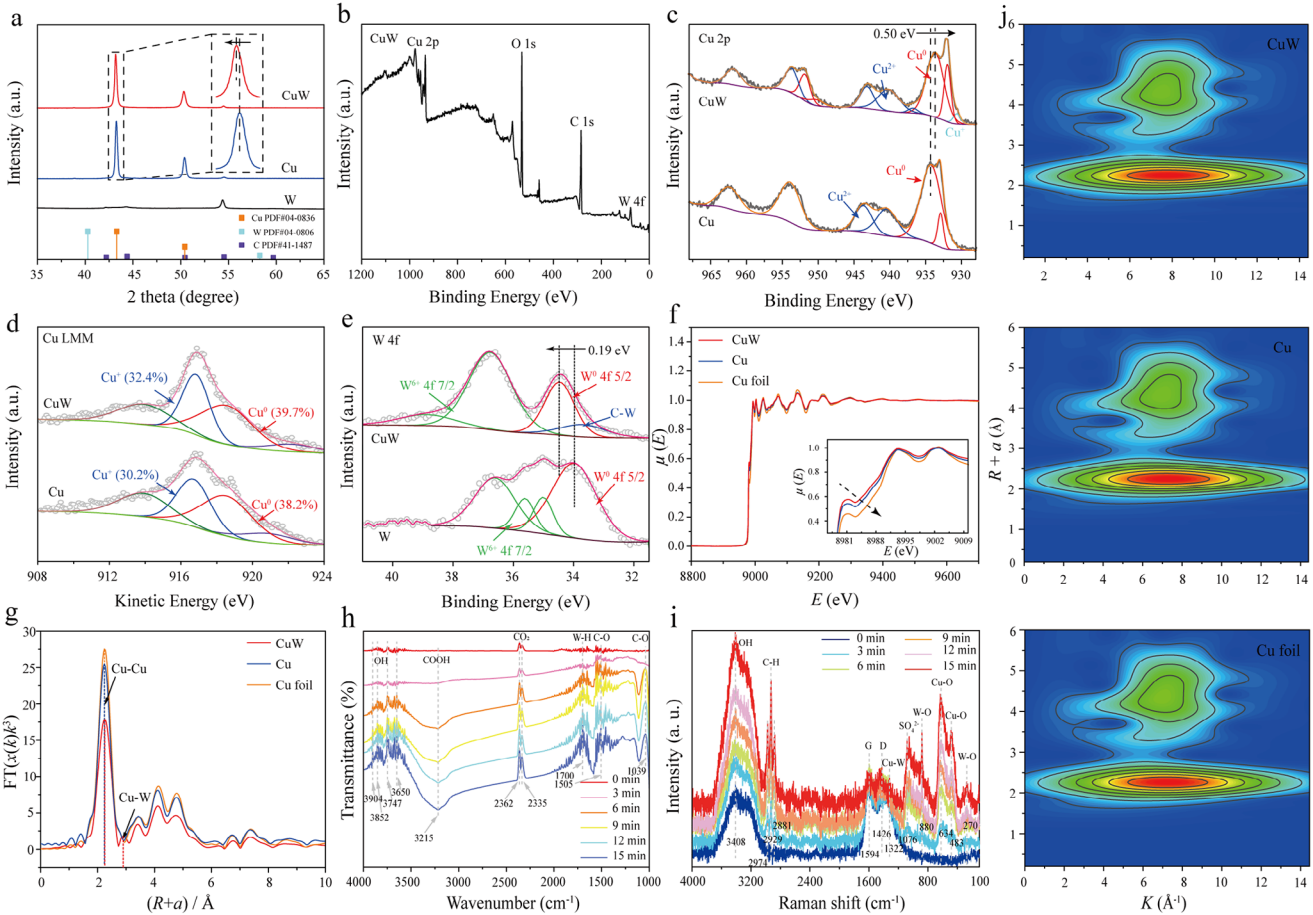
XRD, XPS, XAFS, *in*‐*situ* FT‐IR and *in*‐*situ* Raman data of the materials. a) XRD patterns of CuW, Cu, W. b) XPS survey spectrum of CuW. c–e) High‐magnification XPS spectra of Cu 2p (c) and Cu LMM for CuW and Cu (d). e) XPS spectra of W 4f for CuW and W. f) Cu *K*‐edge XANES spectra. g) *R*‐space extended EXAFS spectra of CuW, Cu, Cu foil. h, i) *In*‐*situ* FT‐IR (h) and *in*‐ *situ* Raman (i) data of the CuW preparation process. j) Wavelet transforms for the *k* ^3^–weighted EXAFS signals of CuW, Cu, and Cu foil.

The crystal structures of the synthesized materials were systematically characterized using X‐ray diffraction (XRD) measurement. As shown in **Figure** **2**a, the XRD pattern confirms that CuW exists as a polycrystalline material, which is further supported by the selected area electron diffraction (HRTEM‐SAED) pattern presented in the inset of Figure 1d. Both pure Cu and W crystallize in a cubic system, with space groups Fm‐3m (225) and Im3m (229), respectively. Compared with Cu comparison material, the (111) and (200) diffraction peaks of CuW prepared under optimal conditions exhibit distinct shifts from 43.30° to 43.18° and 50.36° to 50.43°, respectively. These peak shifts provide clear evidence for the successful doping of W atoms into the Cu lattice, resulting in an expansion of the interplanar distances. Complementary surface chemical analysis was performed using X‐ray photoelectron spectroscopy (XPS). The XPS survey spectrum of CuW reveals the presence of Cu and W elements on the surface of the material (Figure 2b), with Cu and W contents of 96 and 4 at.%, respectively. Detailed examination of the Cu 2p spectrum (Figure 2c) reveals distinct chemical states: peaks at 932.0, 933.7, 950.3, and 951.9 eV correspond to metallic Cu^0^, while peaks at 936.8, 940.2, 943.2, 953.7, and 961.8 eV are attributed to Cu‐O bonds. Interestingly, the Cu^0^ peak in CuW exhibits a negative shift of ≈ 0.50 eV compared to pure Cu, indicating an increased electron density around Cu atoms in the composite material (Figure S10 and Tables S3 and S4, Supporting Information). Further analysis using Auger spectroscopy demonstrates that the concentrations of both Cu^0^ (≈ 918.60 eV) and Cu^+^ (≈ 916.70 eV) species are significantly higher in CuW catalyst compared to the pure Cu catalyst (Figure 2d), suggesting the presence of lower valence state Cu in the CuW nanoalloy. The high‐resolution W 4f spectrum reveals an additional positive shift of 0.19 eV in W^0^ 4f 5/2 binding energy for CuW compared to the pure W catalyst (Figure 2e; Figure S11, Supporting Information), indicating electron transfer from W to Cu atoms. The electron density redistribution from W to Cu in the CuW nanoalloy induces a dual electronic effect: it amplifies the reducibility of Cu while elevating the oxidation propensity of W, as quantitatively supported by the charge transfer metrics in Tables S4 and S5 (Supporting Information). This asymmetric electronic configuration stabilizes the metallic Cu phase and concurrently improves the HOR durability of the nanoalloy. The enhanced stability arises from the synergistic role of WO_3_ species, which not only mitigate Cu oxidation but also provide catalytically active sites for HOR, leveraging the well‐documented HOR activity of tungsten oxides.

To gain deeper insights into the electronic structure and local coordination environment, X‐ray absorption near edge structure (XANES) and extended X‐ray absorption fine structure (EXAFS) analyses were conducted. The *K*‐edge fitting data reveal that CuW exhibits the lowest *E*_CuW_ value at an absorption value *µ*(*E*) of 0.8, indicating superior reducibility of Cu in CuW compared to both pure Cu and Cu foil (Figure 2f; Table S6, Supporting Information).^[^ ^25^, ^26^ ^]^ This enhanced reducibility likely stems from the doping of W atoms into Cu lattice, which increases the electron density around W atoms. EXAFS analysis in *R*‐space demonstrates that the Cu‐W bond length in the nanoalloy is 3.67 Å, while the Cu‐Cu bond length expands to 2.60 Å, compared to 2.56 Å in pure Cu and Cu foil, confirming lattice distortion induced by W doping (Figure 2g). The reduced relative content of Cu in CuW, as indicated by the |*X*(*R*)| values (Figure 2g; Table S7, Supporting Information), suggests that W atoms effectively substitute Cu sites, creating additional structural defects in the nanoalloy.^[^ ^27^ ^]^ The wavelet transform (WT) analysis of the Cu *K*‐edge EXAFS provides further clarification of the coordination environment (Figure 2j). While pure Cu and Cu foil exhibit maximum WT intensity values at 7.3 and 7.2 Å^−1^, respectively, corresponding to Cu‐Cu bonding, the CuW nanoalloy shows a distinct maximum at ≈ 7.8 Å^−1^. This shift in WT intensity maxima suggests the combined influence of both Cu‐Cu and Cu‐W interactions in the composite material, further confirming the successful formation of the CuW alloy structure. The analysis of W *L3*‐edge XANES spectra conclusively demonstrates that Cu‐W alloying induces electron density redistribution from W to Cu. This electronic interaction enhances the reducibility of Cu while elevating the oxidative state of W. At an absorption coefficient *µ*(*E*) = 0.8, the W valence reduction in the CuW alloy relative to pristine W foil (Figure S12a,d,f; Tables S8–S10, Supporting Information) arises from partial W‐O bond formation during alloying. *R*‐space and *K*‐space fitting analyses (Figure S12b–f, Supporting Information) present distinct bonding configurations: the CuW alloy exhibits coexisting Cu‐W, W‐O, and W‐W bonds, whereas the reference W foil primarily contains W‐W bonds with negligible W‐O interactions.^[^ ^28^ ^]^ These findings align with Cu *K*‐edge XANES data, clearly confirming of Cu‐W and W‐O bond formations in the alloy. The observed electronic redistribution mechanism, driven by the higher electronegativity of W (*χ* = 2.36) than that of Cu (*χ* = 1.96). Thus, the pronounced electronegativity of W preferentially drives oxygen coordination, facilitating W‐O bond formation and thereby promoting partial oxidation of W (W^6+^). Concurrently, the relatively lower electronegativity of Cu enables its retention of metallic character (near‐zero oxidation state), which enhances its electron‐donating capacity. This system exhibits a synergistic interplay between two distinct bonding configurations: (1) covalent W‐O interactions (manifested as WO_x_ species) and (2) metallic Cu‐W bonding networks. The coexistence of these bonding modes creates a charge‐compensation mechanism‐the electron‐withdrawing nature of W via W‐O bonds balances with the electron‐donating behavior of Cu through metallic bonding, collectively stabilizing the alloy's electronic structure, which establishes a coherent framework for understanding the alloy's enhanced redox asymmetry and structural stabilization.

To elucidate the synthesis mechanism of CuW nanoalloy, *in*‐*situ* FT‐IR and *in*‐*situ* Raman spectroscopies were employed to monitor the electrode‐solution interface reaction during the preparation of CuW (Figure 2h,i). As the electrodeposition prolongs, the peaks corresponding to Cu‐W (located near 1322 cm^−1^), W‐O (located near 270, 483, 880 cm^−1^), Cu‐O (located near 483, 634 cm^−1^), become gradually intensified.^[^ ^29^, ^30^ ^]^ Concurrently, the D (1350 cm^−1^) and G (1600 cm^−1^) peaks associated with the substrate CP progressively become weakened, indicating the progressively intensified nanoalloy deposition on CP, thereby covering the substrate more extensively.^[^ ^31^, ^32^ ^]^ Significantly, the emergence of C‐H species bonding on the catalyst surface becomes more pronounced at prolonged electrodeposition, demonstrating that sodium citrate, acting as a chelating agent, has participated in the electrodeposition process.^[^ ^33^ ^]^ This chelate is beneficial for controlling the nanoscale dimensions of the alloy. Additionally, the OH peak (3408 cm^−1^) becomes increasingly prominent, suggesting that OH^‒^ and water molecules play a crucial role in the deposition process, including ion diffusion, transfer, and deposition reduction reactions.^[^ ^34^ ^]^ Furthermore, the adsorption of hydrogen on the electrode surface implies that a small amount of hydrogen gas may be generated during the preparation process, which is conducive to the formation of porosity and nanostructure on the alloy surface. Additionally, *in*‐*situ* FT‐IR analysis reveals that with the increasing deposition time, the amount of OH adsorption (3650–3904 cm^‒1^) is gradually increased, the COOH bond (3215 cm^‒1^) becomes significantly stronger, and the W‐H bond (1700 cm^‒1^) intensity is also progressively enhanced, confirming the gradually elevated W amount in the nanoalloy.^[^ ^35^, ^36^, ^37^ ^]^ These findings are consistent with the *in*‐*situ* Raman results.

### 2.2 The HOR Performance of CuW

**Figure 3:**
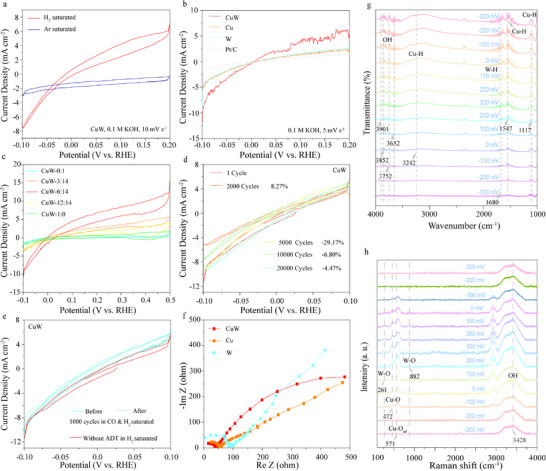
HOR electrocatalytic performances of the CuW catalyst. a) CV curves of CuW measured in H_2_ or Ar‐saturated 0.1 m KOH electrolytes. b) LSV curves of CuW, Cu, W, and Pt/C in H_2_‐saturated 0.1 M KOH electrolyte. c) HOR performances of CuW prepared by the same synthesis method but with different Cu^2+^/WO_3_^2−^ molar ratios. d) ADT curves of CuW. e) Tolerance test data of CuW to CO poisoning before and after 1,000 cycles of ADT in the 1,000 ppm CO/H_2_‐saturated 0.1 m KOH electrolyte. f) EIS data of CuW, Cu, and W at open‐circuit potential in 0.1 m KOH electrolyte. g, h) *In*‐*situ* FT‐IR (g) and *in*‐*situ* Raman (h) results of CuW HOR catalytic process.

The HOR performances of the materials were evaluated in a standard three‐electrode system using H_2_‐saturated 0.1 m KOH electrolyte at 25 °C. Prior to the measurements, the reversible hydrogen electrode (RHE) calibration is executed using cyclic voltammetry (CV) in the same system (Figure S13, Supporting Information). The working electrode was a self‐supporting material, while a carbon rod served as the counter electrode and HgO/Hg (in 1.0 m KOH solution) as the reference electrode. To assess the HOR activities of the CuW catalyst, CV measurements were conducted in both Ar‐saturated and H_2_‐saturated electrolytes. Notably, the CuW shows significantly higher current density in the H_2_‐saturated electrolyte compared to the CP substrate (**Figure** **3**a; Figure S14c, Supporting Information), revealing its superior HOR activity. Linear sweep voltammetry (LSV) data reveal that the CuW achieves the highest H_2_ oxidation current density (1.86 mA cm^−2^) at 0.05 V vs. RHE, outperforming both Cu and W catalysts (Figure 3b), indicating the doping with W atoms greatly enhances the HOR activity of the catalyst. Further analysis of the micro‐polarization region of the polarization curve through linear fitting yielded the exchange current density (*j*_0_), which is positively correlated with the kinetic current density. The *j*_0_ value of CuW (1.15 mA cm^−2^) is substantially higher than those of Cu (0.50 mA cm^−2^) and W (0.51 mA cm^−2^), further confirming that W doping effectively improves the activity of the catalyst and shows a good HOR performance (Figure 3b; Table S11, Supporting Information). Hydrogen temperature‐programmed desorption (H_2_‐TPD) was employed to quantify hydrogen spillover capacity. At 140 °C, the H₂‐TPD measurements reveal hydrogen spillover amounts of 15, 15, and 6 *µ*mol g⁻¹ for CuW alloy, pure Cu, and pure W, respectively. These results not only confirm the enhanced hydrogen spillover efficiency of the CuW alloy but also suggest its potential to facilitate HOR kinetics (Figure S15, Supporting Information).^[^ ^38^ ^]^ The turnover frequencies (TOFs) for CuW, Cu, and W are measured to be 1.03, 0.44, 0.94 s^−1^, respectively, at an overpotential of 200 mV (Figure S16, Supporting Information).^[^ ^39^ ^]^ Additionally, the influence of electrodeposition conditions on the HOR electrocatalytic performance of the CuW catalyst was investigated using CV. As illustrated in Figure 3c and Figure S14a,b (Supporting Information), the CuW catalyst prepared with a Cu^2+^ to WO_3_^2−^ ratio of 6:14 in the reactant solution, a deposition temperature of 70 °C, and a deposition time of 15 min exhibits the highest HOR performance. This result aligns with the SEM characterization under optimal conditions, which reveals the highest density of active sites on the catalyst surface.

In addition to evaluating the HOR activity, the stability of CuW was assessed through an accelerated durability test (ADT) conducted over a potential range of −0.1–0.1 V vs. RHE for 20, 000 cycles. As depicted in Figure 3d, the current density at 0.05 V vs. RHE remains nearly unchanged after 20,000 cycles, even surpassing the performance of commercial Pt/C (Figure S17a, Supporting Information). The evolution of the CuW catalyst's morphology and chemical state during long‐term cycling is critical to understanding its HOR performance stability. Post‐ADT characterization reveals no significant changes in the morphology, lattice spacing, crystal structure, elemental composition, or stoichiometry of the CuW catalyst (Figures S18–S22 and Tables S12–S14, Supporting Information), underscoring its exceptional durability in alkaline environments. XPS analysis of the CuW nanoalloy after ADT (CuW‐ADT) reveals that the binding energies of Cu and W shifted to higher levels compared to their original states, suggesting the formation of amorphous oxides on the CuW surface after 20,000 cycles, which increases the material's surface oxidation. This phenomenon may enhance the catalyst's ability to oxidize hydrogen, contributing to its improved performance. Furthermore, the CO tolerance of the CuW catalyst is evaluated using CV ADT method in a 1,000 ppm CO/H_2_−saturated 0.1 m KOH solution and performed over 1,000 cycles within a potential range of −0.1–0.5 V vs. RHE. Remarkably, the current density in the CO‐containing environment is significantly higher than that in a pure H_2_ environment, with no noticeable degradation (Figure 3e). Based on the *in*‐*situ* FT‐IR spectra in Figure S23d (Supporting Information), it can be observed that CO (2122, 1213 cm⁻¹) adsorbs on the CuW surface, and under alkaline conditions, it is oxidized to form COO⁻ (1642 cm⁻¹) and CO_2_ (2352 cm⁻¹). This indicates that the catalyst effectively mitigates CO poisoning by converting adsorbed CO into non‐toxic species.^[^ ^36^, ^37^ ^]^ In alkaline electrolytes, the electrochemical oxidation of CO follows the equations below:

Overall reaction:

(1) $$\mathrm{CO}+2{\mathrm{OH}}^{-}\to {{\mathrm{CO}}_{3}}^{2-}+H_{2}O+2e^{-}$$

Intermediate steps:

(2) $$\mathrm{CO}\;\mathrm{adsorption}:\mathrm{CO}+*\;\to {\mathrm{CO}}_{\mathrm{ads}}\left(*=\mathrm{catalytic}\;\mathrm{active}\;\mathrm{site}\right)$$

(3) $${\mathrm{OH}}^{-}\;\mathrm{adsorption}:{\mathrm{OH}}^{-}+*\;\to {\mathrm{OH}}_{\mathrm{ads}}+e^{-}$$

CO oxidation (Langmuir‐Hinshelwood mechanism):

(4) $${\mathrm{CO}}_{\mathrm{ads}}+{\mathrm{OH}}_{\mathrm{ads}}\to \mathrm{COO}{H_{\mathrm{ads}}}^{-}+*$$

Carboxylate intermediate decomposition:

(5) $$\mathrm{COO}{H_{\mathrm{ads}}}^{-}+{\mathrm{OH}}^{-}\to {{\mathrm{CO}}_{3}}^{2-}+H_{2}O+e^{-}$$

CO severely inhibits hydrogen spillover by blocking H adsorption sites, disrupting H migration to supports, and reducing support activation because it binds strongly compared to H. This leads to lower catalytic efficiency in hydrogen spillover. However, this excellent CO tolerance can be attributed to the occurrence of CO oxidation reactions on its surface, highlighting its potential for practical applications in CO‐contaminated environments.

Electrochemical impedance spectroscopy (EIS) was utilized to analyze the electrochemical resistance characteristics of the synthesized materials. The EIS curves of prepared CuW, Cu, and W are obtained at the open‐circuit potential in 0.1 M KOH electrolyte (Figure 3f). The corresponding equivalent circuit diagram along with the fitting results are shown in Figure S11b (Supporting Information). Notably, the CuW demonstrates significantly lower charge transfer resistance (*R*_ct_, 34.8 Ω) at the electrolyte/electrode interface values, compared to that of Cu and W (Table S15, Supporting Information). This reduced *R*_ct_ value indicates enhanced charge transfer kinetics in the CuW catalyst, which correlates with its superior HOR activity.

### 2.3 The HOR Catalytic Mechanism of CuW

To gain deeper insights into the origin of the high HOR catalytic activity of CuW catalyst, *in*‐*situ* FT‐IR and *in*‐*situ* Raman were employed to monitor the dynamic behavior of H and OH species on the surface of CuW catalyst during the HOR process. As depicted in Figure 3g, the peak intensities of OH, Cu‐H, and W‐H species on the surface of CuW increase as the potential shifts positively from −0.3 to 0 V, confirming that the reactants OH and H species, essential for the HOR process, are progressively enriched on the catalyst surface within this potential range as the voltage increases.^[^ ^35^, ^37^, ^40^, ^41^, ^42^, ^43^ ^]^ When the potential is further shifted positively from 0 to 0.3 V and then returned to 0 V, the intensities of the OH, Cu‐H, and W‐H peaks remain almost constant. This observation suggests that a dynamic equilibrium has been established between the continuous consumption of H and OH species by the HOR reaction and their sustained replenishment in the surface layer of the CuW catalyst. Conversely, when the potential drops from 0 to −0.3 V, the peak intensities of OH, W‐H, and Cu‐H species gradually increase, corresponding to the termination of the HOR process, which leads to the accumulation of these species on the catalyst surface.

The *in*‐*situ* Raman spectra of CuW reveal distinct characteristic peaks at W‐O (261, 882 cm^−1^), Cu‐O_ad_ (571 cm^−1^) and Cu‐O (472 cm^−1^) under an applied potential of 0.3 V vs. RHE (Figure 3h).^[^ ^44^, ^45^ ^]^ These observations are consistent with the spectral features of the freshly prepared CuW sample, confirming the adsorption of OH species on the catalyst surface at this potential. As the potential decreases, these characteristic peaks become gradually diminished and eventually disappear at a potential of −0.1 V vs. RHE, indicating that the implantation of hydrogen ions into the W/WO_3_ substrate, thereby confirming the occurrence of hydrogen spillover effect, which facilitates the desorption of ^*^H species from W/WO_3_ and the subsequent re‐exposure of the W/WO_3_ surface. Upon applying a positive potential sweep (−0.3–0.3 V), these Raman characteristic peaks reappear, indicating that the previously injected H ions into the substrate have either been withdrawn or reacted with ^*^OH to generate H_2_O.

**Figure 4:**
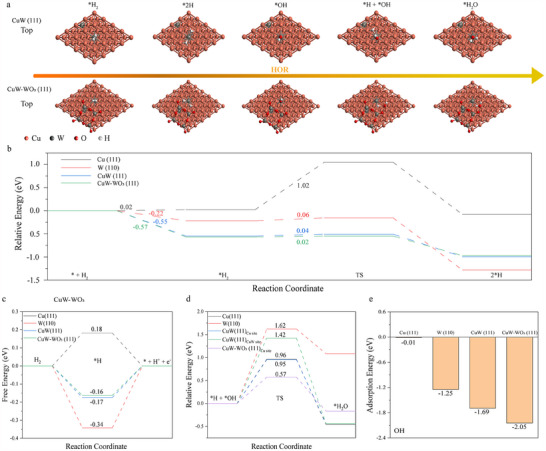
a) Schematic illustration of HOR catalytic process. b) The calculated adsorption energy and dissociation barrier of H_2_ on Cu (111), W (110), CuW (111) and CuW‐WO_3_ (111) surfaces. c) The calculated free energy diagram for H adsorption energy on Cu (111), W (110), CuW (111), and CuW‐WO_3_ (111) surfaces. d) Minimum energy pathways of HOR on the surface of Cu (111), W (110), CuW (111)_Cu site/CuW site_, and CuW‐WO_3_ (111)_Cu site_ surfaces. e) Adsorption energies of OH on Cu (111), W (110), CuW (111), and CuW‐WO_3_ (111) surfaces.

Density functional theory (DFT) calculations were conducted to gain an in‐depth understanding of the fundamental mechanism underlying HOR catalyzed by CuW alloy in alkaline media. Detailed calculations and parameters have been provided in Supplementary data. The Cu (111), W (110), CuW (111), and CuW‐WO_3_ (111) were selected as model surfaces, as these were the preferentially exposed lattice planes of Cu, W, CuW and CuW‐WO_3_ nanoalloys, respectively, based on the XRD and CLAC‐STEM data (Figures S24–S27, Supporting Information). **Figure** **4**a schematically illustrates the key steps of HOR process on CuW nanoalloy or CuW‐WO_3_ surface, including H_2_ adsorption, decomposition, and the formation of H_2_O through the reaction of ^*^H species with ^*^OH species. To quantitatively evaluate the HOR activity, the adsorption energy, dissociation, and oxidation barriers of H_2_ were calculated. As shown in Figure 4b, the adsorption energies of ^*^H_2_ are 0.02, −0.22, −0.55, −0.57 eV, and the dissociation barrier of H_2_ are 1.02, 0.06, 0.04, and 0.02 eV for Cu (111), W (110), CuW (111), and CuW‐WO_3_ (111), respectively. Therefore, the adsorption and dissociation of H_2_ are highly favorable on W (110) as compared to Cu (111), and meanwhile, introduction of W can strengthen H_2_ adsorption and promote H_2_ dissociation on CuW‐WO_3_ (111) (Tables S16 and S17, Supporting Information). The free energies of hydrogen adsorption (*ΔG*_H_) are 0.18, −0.34, −0.17, and −0.16 eV for Cu (111), W (110), CuW (111), and CuW‐WO_3_ (111), respectively, suggesting that H is the most easily adsorbed on CuW‐WO_3_ (111) as a spontaneous process (Figure 4c; Table S18, Supporting Information). In terms of ^*^OH species adsorption in an alkaline environment, Cu (111) (‐0.01 eV), W (110) (‐1.25 eV), CuW (111) (‐1.69 eV) and CuW‐WO_3_ (111) (‐2.05 eV) exhibit spontaneous adsorption of ^*^OH species (Figure 4e; Table S19, Supporting Information), implying the increased adsorption capacity of the catalyst for OH species by W doping and the formation of WO_3_. The oxidation of ^*^H species into H_2_O (^*^H + ^*^OH = H_2_O) on Cu (111) and W (110) needs to overcome the energy barriers of 0.96 eV and 1.62 eV, respectively (Figure 4d; Table S20, Supporting Information), indicative of the much slower oxidation kinetics over W (110) than over Cu (111). Therefore, HOR activities of W (110) and Cu (111) are respectively dependent on H_2_ adsorption/ dissociation, OH adsorption and ^*^H oxidation process (Figure S28, Supporting Information).

To further elucidate the role of the CuW alloy, ^*^H oxidation on two different sites of CuW (111), i. e., CuW_cu site_ and CuW_W site_, are considered. The energy barrier of ^*^H oxidation to H_2_O is 0.95 eV on Cu sites, which is significantly lower than that on W sites (1.42 eV), meaning that the ^*^H oxidation predominantly occurs on the Cu sites of CuW. The energy barrier of ^*^H oxidation to H_2_O is 0.57 eV on CuW‐WO_3_ (111)_cu site_. Thus, these findings collectively demonstrate that the high HOR catalytic activity of CuW catalyst arises from the efficient adsorption and dissociation of H_2_ on the W/WO_3_ sites, followed by the rapid transport of generated ^*^H to the Cu site through a hydrogen spillover effect, where it reacts with ^*^OH species and completes the oxidation process. Furthermore, the thermodynamic stability of W‐doped Cu supercell was systematically investigated through formation energy analysis. The substitutional formation energy *ΔE*(formation) for replacing a Cu atom with W was calculated using the thermodynamic expression:

(6) $$\Delta E\left(\mathrm{formation}\right)=E\left(\mathrm{doped}\right)-E\left(\mathrm{pure}\right)+n\left(\mu_{\mathrm{Cu}}-\mu_{W}\right)$$

The *E*(doped) and *E*(pure) represent the total energies of the doped and pristine supercells, respectively. The parameter *n* denotes the number of substitution events, while *µ*_Cu_ and *µ*_W_ correspond to the chemical potentials of Cu and W under periodic boundary conditions, their values being constrained by synthesis‐dependent boundary conditions. These chemical potentials were calculated by calculating the energies of isolated W and Cu atoms within equivalent computational frameworks.^[^ ^46^ ^]^ The computed formation energy for single W substitution in Cu yields a negative value of −2.981 eV (Table S21, Supporting Information). This thermodynamically favorable energy landscape indicates that W incorporation into the Cu matrix is energetically preferred, suggesting enhanced experimental feasibility for fabricating CuW nanoalloy.

Building on the high hydrogen spillover capability and strong hydrogen adsorption properties of CuW catalyst, diverse catalytic applications are successfully extended, including methanol oxidation reaction (MOR) under alkaline conditions, where methanol is efficiently oxidized to carbonate ions (Figure S29, Supporting Information). Furthermore, the catalyst achieves robust HOR performance across a broader pH range, spanning acidic to neutral environments (Figure S23, Supporting Information). Owing to its simple, environmentally benign synthesis method, CuW retains its inherent crystallinity and maintains excellent HOR activity even when scaled up for large‐scale production (Figure S30, Supporting Information). Consequently, with further optimization of industrial‐scale manufacturing processes, CuW holds significant promise for widespread applications (Figure S30, Supporting Information).

## 3 Conclusion

In summary, we have successfully developed a facile and high‐efficiency electro‐deposition approach to *in*‐*situ* synthetize CuW (∼5 nm) HOR catalyst using CP as the substrate. The uniform introduction of W atoms into Cu lattice promotes the generation of numerous heterointerfaces, leads to the shift of electron cloud from W to Cu and forming WO_3_. The as‐prepared CuW shows an excellent HOR performance with significantly high activity (1.86 mA cm^−2^ at 0.05 V vs. RHE) and exchange current density (1.15 mA cm^−2^). By integrating experimental results with theoretical calculations, we reveal that high HOR activity of CuW stems from the hydrogen spillover effect between abundant heterogeneous interfaces, as well as the synergistic enhancement of catalytic properties: W/WO_3_ sites exhibit superior activity for H_2_ adsorption and dissociation, while Cu sites show enhanced capability for HOR. This study provides an innovative strategy for designing highly efficient PGM‐free HOR catalysts with abundant metal‐metal/ metallic oxide heterointerfaces for electrocatalysis.

## Conflict of Interest

The authors declare no conflict of interest.

## Author Contributions

J. S. and B. X. led the project. B. X. designed and performed the experiments. B. X., L. C., and J. S. wrote the manuscript. All authors discussed the results and commented on the manuscript.

## Supporting information



Supporting Information

## Data Availability

The data that support the findings of this study are available from the corresponding author upon reasonable request.
