# Supplementary material for: Electrocatalytic Hydrogen Oxidation by Defect‐Enriched CuW Nanoalloy Featuring Hydrogen Spillover Effect
Source: Adv Sci (Weinh). 2025 Jun 20;12(34):e03710. doi: 10.1002/advs.202503710 (PMC12442599; doi:10.1002/advs.202503710)
Supplement: Supplementary file 1 — Supporting Information [file ADVS-12-e03710-s001.docx]

# Supporting Information

**Electrocatalytic hydrogen oxidation by defect-enriched CuW nanoalloy featuring hydrogen** **spillover effect**

*Bingyan Xiong**,*,* *Lisong Chen, Jianlin Shi**

Dr. B. Xiong

School of Medicine, Tongji University, Shanghai, 200331, P. R. China

E‒mail: xiongbingyan720@126.com

Prof. L. Chen

Shanghai Key Laboratory of Green Chemistry and Chemical Processes, School of Chemistry and Molecular Engineering, East China Normal University, Shanghai 200062, P. R. China

Prof. J. Shi

School of Medicine, Tongji University, Shanghai, 200331, P. R. China

Shanghai Institute of Ceramics Chinese Academy of Sciences, Shanghai, 200050, P. R. China

E-mail: jlshi@mail.sic.ac.cn

**Experimental Section**

*Materials*: All chemicals used in this work were as received without any further purification. Cupric sulfate anhydrous (CuSO_4_, Analytical Reagent ≥ 99.0%) was purchased from Macklin. Sodium tungstate dihydrate (Na_2_WO_4_^.^2H_2_O, Reagent Grade ≥ 98%), sodium citrate dihydrate (C_6_H_5_Na_3_O_7_^.^2H_2_O, Analytical Reagent ≥ 99.0%), sodium bromide (NaBr, Analytical Reagent ≥ 99.0%) were purchased from Adamas. Potassium hydroxide (KOH, Analytical Reagent ≥ 85%) was purchased from Sinopharm Chemical Reagent. Hydrogen (H_2_, 99.999%) and Nitrogen (N_2_, 99.999%) were purchased from PUJIANG.

*Preparation of Electrodes*: CuW, this self-supporting electrode material was synthesized by electro-deposition method. In detail, for the pretreatment of carbon paper (1 cm × 1 cm), the isopropanol, deionized water, 0.1 M HCl and deionized water were employed as sonication solvents for 30 minutes, respectively. The time for pretreatment of electroplate liquid was 30 min, it was contained 0.06 M CuSO_4_, 0.14 M Na_2_WO_4_^.^2H_2_O, 0.5 M Na_3_C_6_H_5_O_7_^.^2H_2_O, 0.15 M NaBr at 70ºC under medium-speed stirring with N_2_-bubbled to keep a N_2_-saturated environment until the end of synthesis. The cyclic voltabsorptometry cycles were performed in the potential range of -2.1 V to -0.6 V vs. Ag/AgCl at a scan rate 100 mV/s, while fixed at -2.1 V and -0.6 V for 0.05 s and 0.5 s in each cycle, respectively, and the whole electro-deposition time was 15 minutes.

Cu, the fabricate conditions were similar with CuW, except that Na_2_WO_4_^.^2H_2_O was not added in electroplate liquid.

W, the fabricate conditions were similar with CuW, except that CuSO_4_ was not added in electroplate liquid.

Pt/C, the catalyst ink was contained 0.0010 g 20 wt% Pt/C, 2378 *μ*L alcohol, 8267 *μ*L water and 679 *μ*L 5 wt % Nafion, then ultrasonication for 30 min to get a uniform solution. 142 *μ*L catalyst ink was transferred by a pipette onto a pre-cleaned carbon paper (1 cm*1 cm) and formed a mass loading of 2.5 *μ*g_Pt_ cm^−2^. Then, the electrode material was dehydrated at room temperature before electro-chemical measurements.

*Structural Characterization*: The Hitachi S-4800 scanning electron microscope with a 3 kV accelerating voltage was employed to collect SEM images. The JEOL-2010F electron microscope operated at 200 kV was hired to obtain HRTEM and EDS data. The Thermofisher Spectra 300 Condenser Lens Aberration-Corrected Scanning Transmission Electron Microscope (CLAC-STEM) operated at 300 kV with superXG2 Energy-Dispersive X-ray Spectrometer (EDS) was employed to investigate the atomic-scale structure of the material. The Rigaku D/Max-2550 V X-ray diffractometer with Cu-K*α* radiation target (40 kV, 40 mA, *λ*= 1.5418 Å) was used to get XRD data. The Thermo Fisher Scientific, ESCALAB250Xi instrument was applied to acquire XPS results with monochromatic Mg Ka X-rays at 1253.6 eV and operating at 150 W. The C 1s electron peak (BE = 284.8 eV was the internal reference for the spectrum. Cu *K*-edge XANES data of the CuW, Cu, Cu foil were measured in the fluorescence mode at the BL14W1 beam line of the Shanghai Synchrotron Radiation Facility (SSRF), China. W *L3*-edge XANES data of the CuW, W, W foil were measured by EasyXafs300+. According to the standard procedures, the test results were analyzed. The structural characterization of liquid products was performed by nuclear magnetic resonance (NMR) spectroscopy. Both ^1^H and ^13^C NMR spectra were recorded on a Bruker Avance II 500 high-field spectrometer (operating at 500 MHz/125 MHz).

*Electrochemical Measurement*: The Bio-Logic VSP-300 electro-chemical system was used to perform the HOR measurements at 25°C. The working electrode was the self-supporting material, the graphite was the auxiliary electrode and the HgO/Hg (filled with 1.0 M KOH solution) was the reference electrode. The reference of the potentials occurred in this work was reversible hydrogen electrode (RHE). And the 25°C H_2_-saturated 0.1 M KOH solution was the electrolyte used in this work. The LSV measurements were performed in the potential range of -0.1 to 0.2 V vs RHE at a scan rate of 5 mV s^-1^. The CV measurements were carried out in the potential range of -0.1 to 0.1/0.2/0.5 V vs RHE at a scan rate of 5 mV s^-1^. The RDE measurements were performed by RRDE-3A, and the rotate speed was 1, 600 rpm in room temperature. The EIS measurements were implemented in the frequency range of 2×10^6^ to 0.1 Hz at the open circuit potentials.

*RHE Calibration*: The RHE calibration was executed in a typical three electrodes system by the CV measurement with H_2_-saturated 0.1 M KOH electrolyte at the temperature of 25°C. The platinum-wire electrodes were employed as the working electrode and the counter electrode. And the reference electrode was the researched HgO/Hg (filled with 1.0 M KOH solution). When the current value was zero, the mean value of the potentials was obtained as the reference electrode potential (Figure S13). HgSO_4_/Hg and HgO/Hg reference electrodes were also tested in pH 1 and pH 7 electrolytes (Figure S23).

**
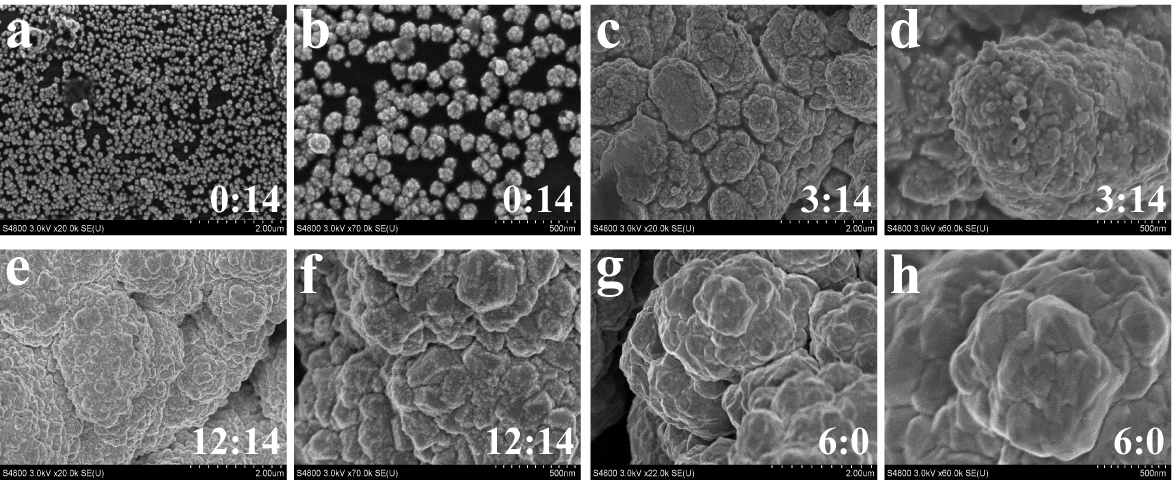
Figure S1** SEM images of samples. a, b) Cu:W=0:14, c, d) Cu:W=3:14, e, f) Cu:W=12:14, g, h) Cu:W=6:0 synthesized with varied molar ratio of reactants (Cu^2+^ and WO_3_^2-^) but with the same fabricated method.

**
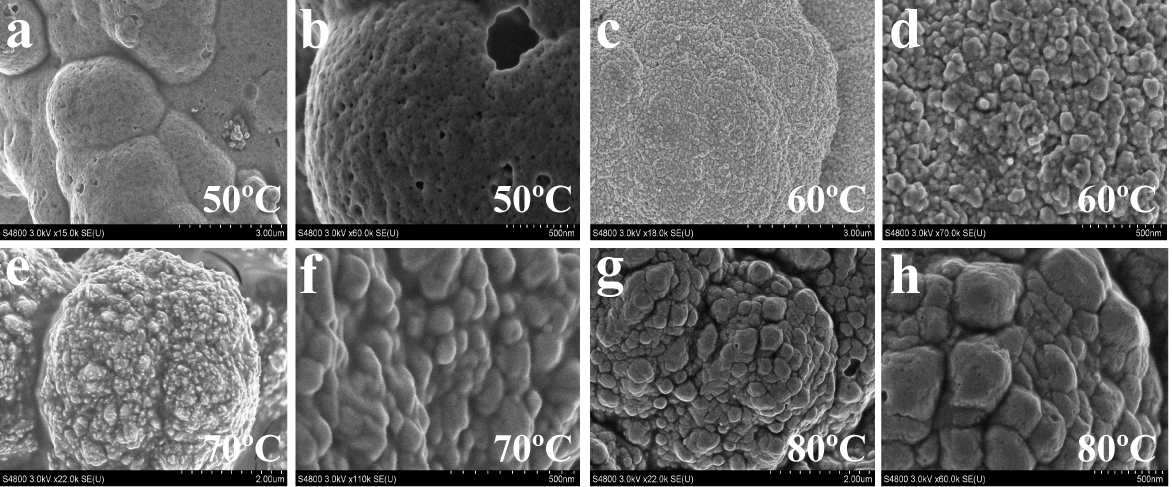
****Figure S2** SEM images of samples. Materials were synthesized with varied temperatures a, b) 50ºC, c, d) 60ºC, e, f) 70ºC, g, h) 80ºC but with the same fabricated method.


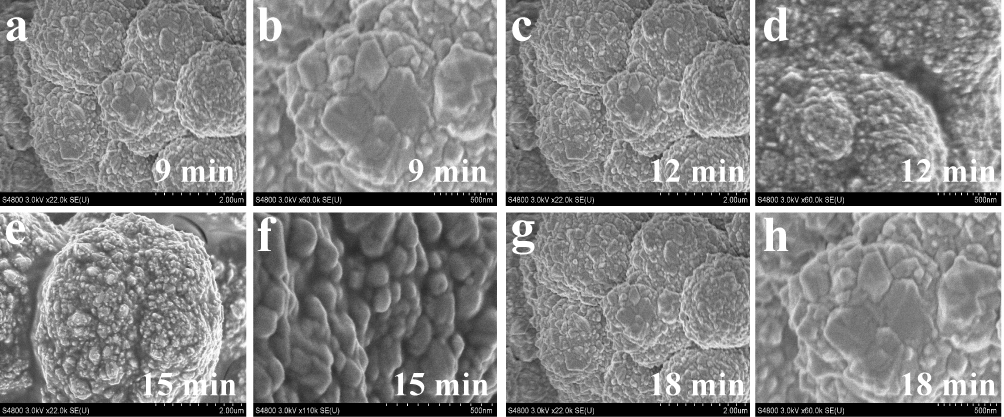


**Figure S3** SEM images of samples. Materials were synthesized with varied time a, b) 9 min, c, d) 12 min, e, f) 15 min, g, h) 18 min but with the same fabricated method.


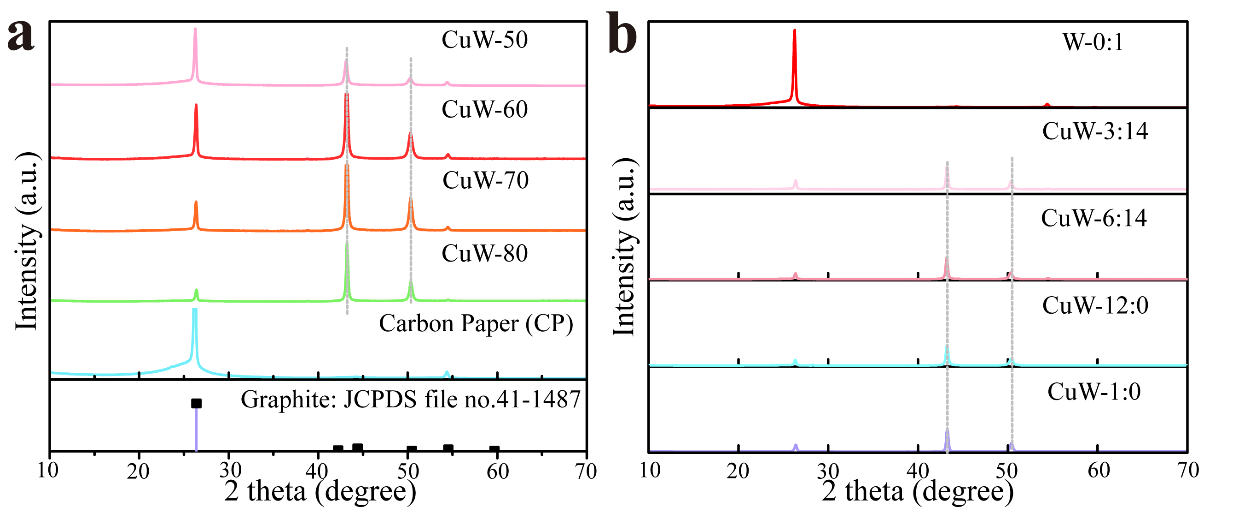


**Figure S4** XRD data of materials. XRD data for the materials synthesized with a) different electro-deposition temperature (50ºC, 60ºC, 70ºC, 80ºC) and b) various molar ratios of Cu and W (0:1, 3:14, 6:14, 12:0, 1:0).

**
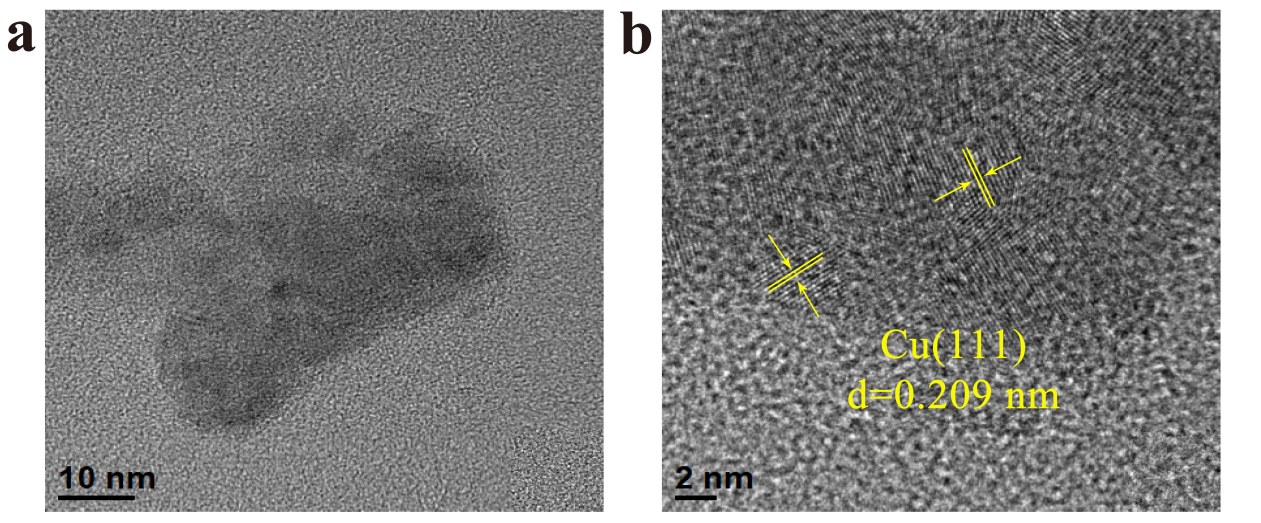
**

**Figure S5** a) TEM and b) HRTEM images of Cu.

**
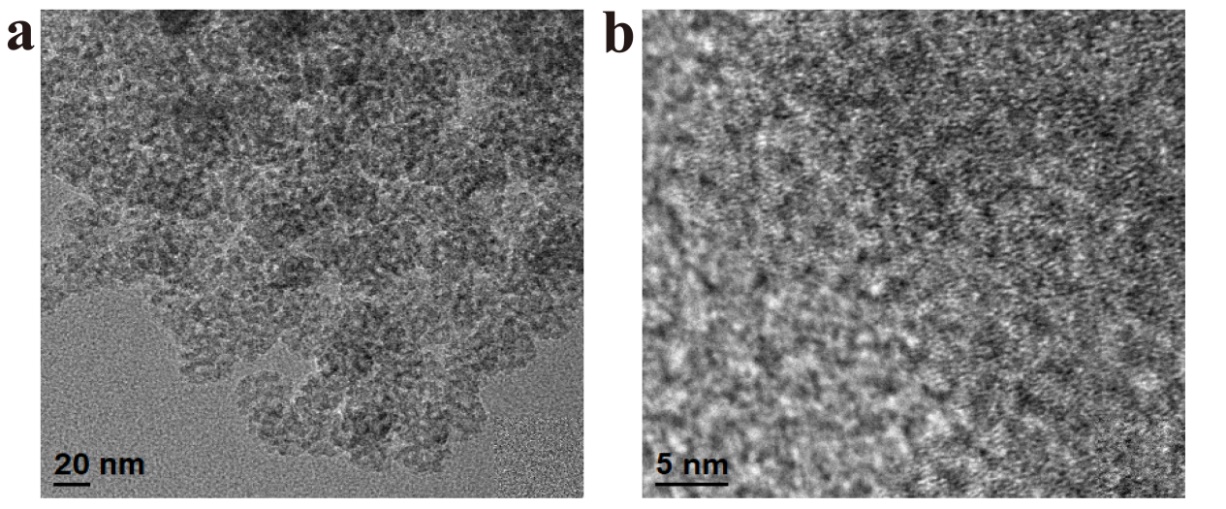
**

**Figure S6** a) TEM and b) HRTEM images of W.


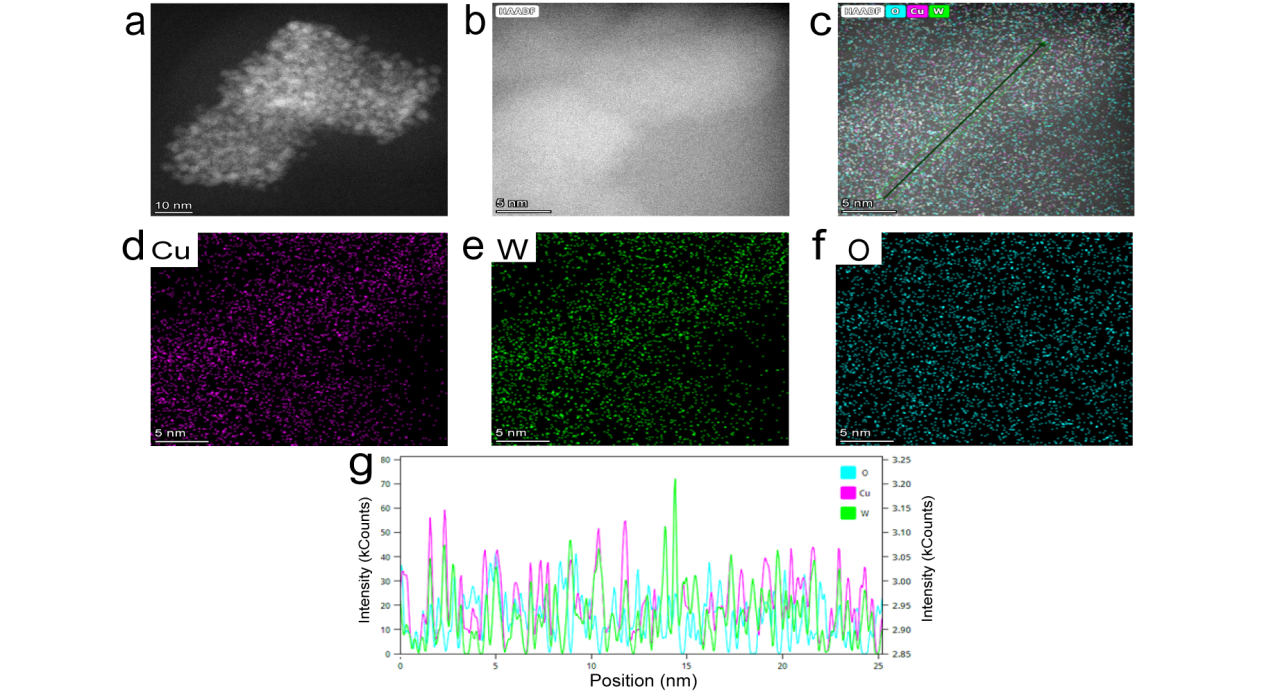


**Figure S7** The CLAC-STEM characterization of the CuW sample. a) Dark-field HADDF image, b) bright-field HADDF image, c) EDS composite map marking the line-scan region (g), and d-f) elemental mapping images for Cu (d), W (e), and O (f).


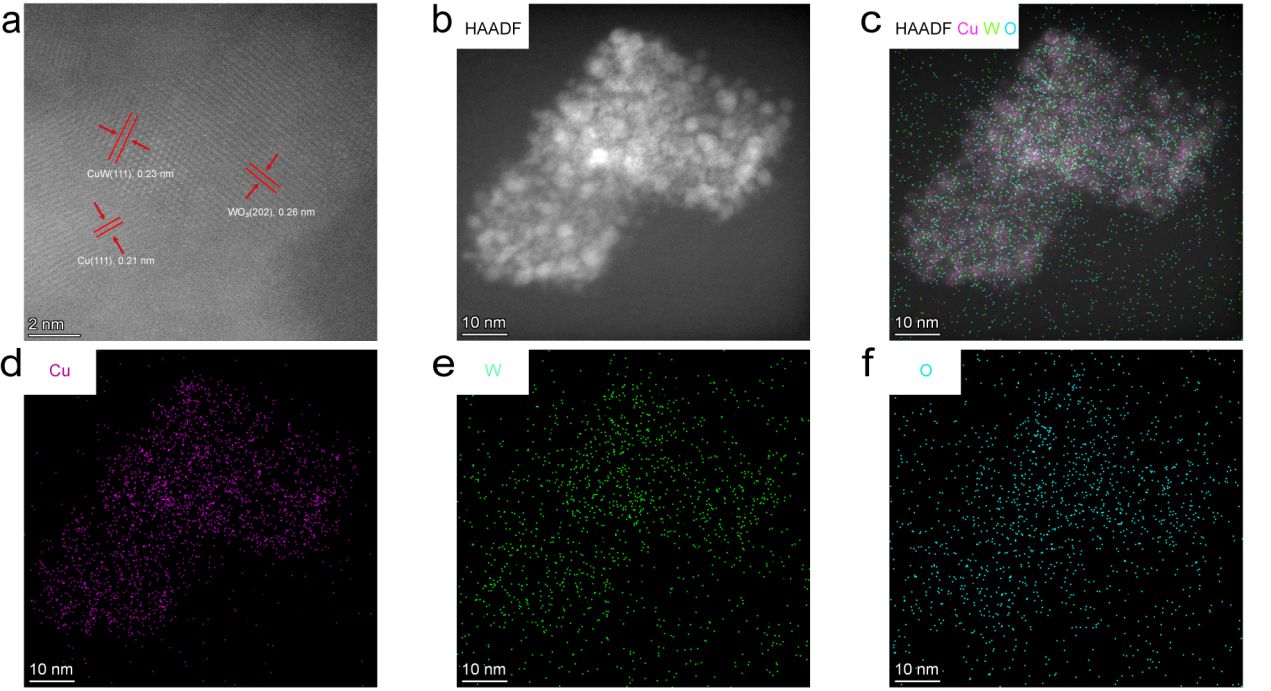


**Figure S8** High-resolution CLAC-STEM images of CuW. a) Spherical aberration-corrected high-resolution lattice fringe analysis. b) HAADF image. c) EDS elemental mapping. d) EDS elemental mapping for Cu. e) EDS elemental mapping for W. f) EDS elemental mapping for O.


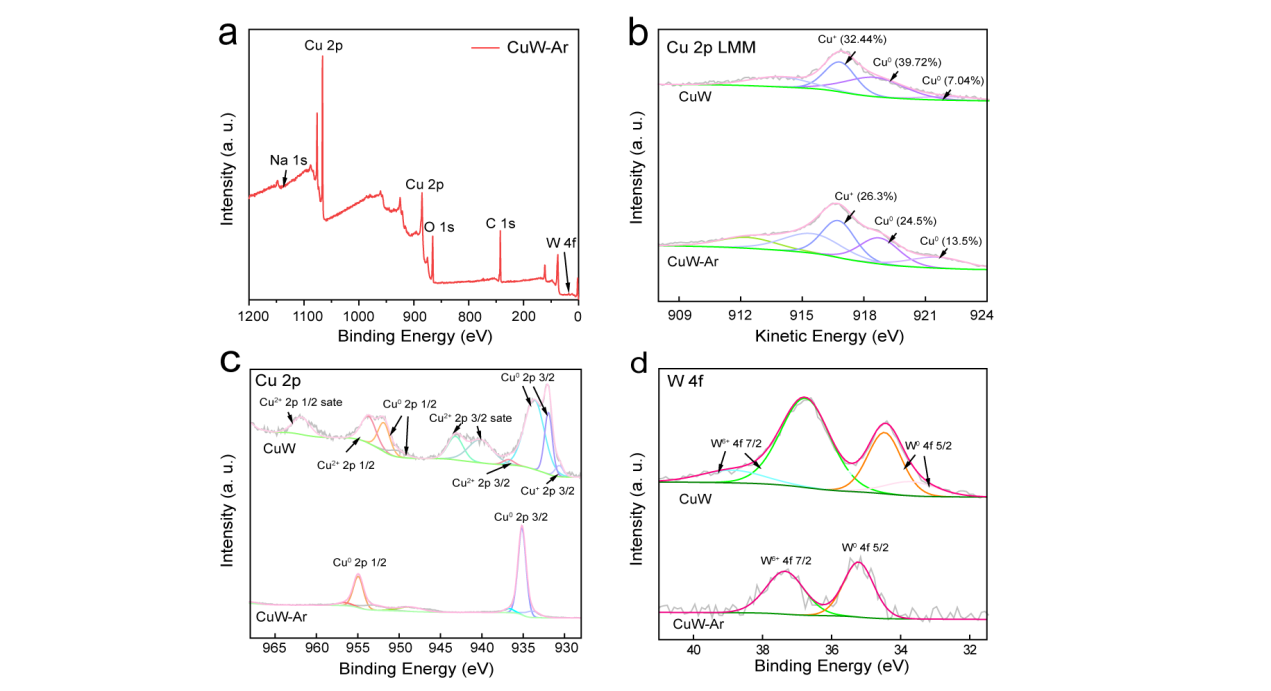


**Figure S9** XPS spectra of CuW sample after argon plasma etching. a) Wide-scan spectrum, b) Cu 2p LMM Auger spectra, c) Cu 2p spectra, d) W 4f spectra.


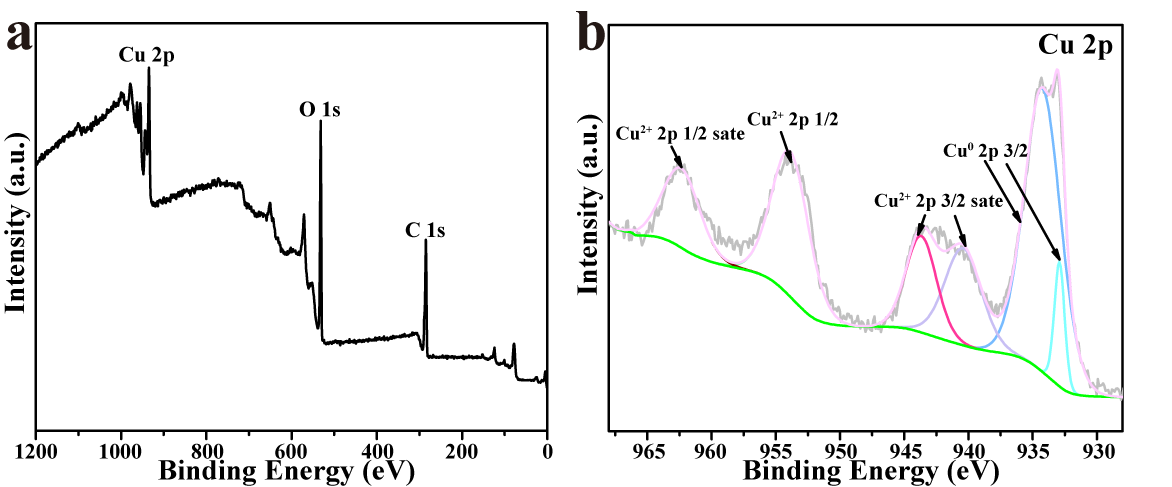


**Figure S10** XPS data of Cu. a) XPS survey spectrum and b) the high-resolution XPS spectrum of Cu 2p from the contrast sample Cu.


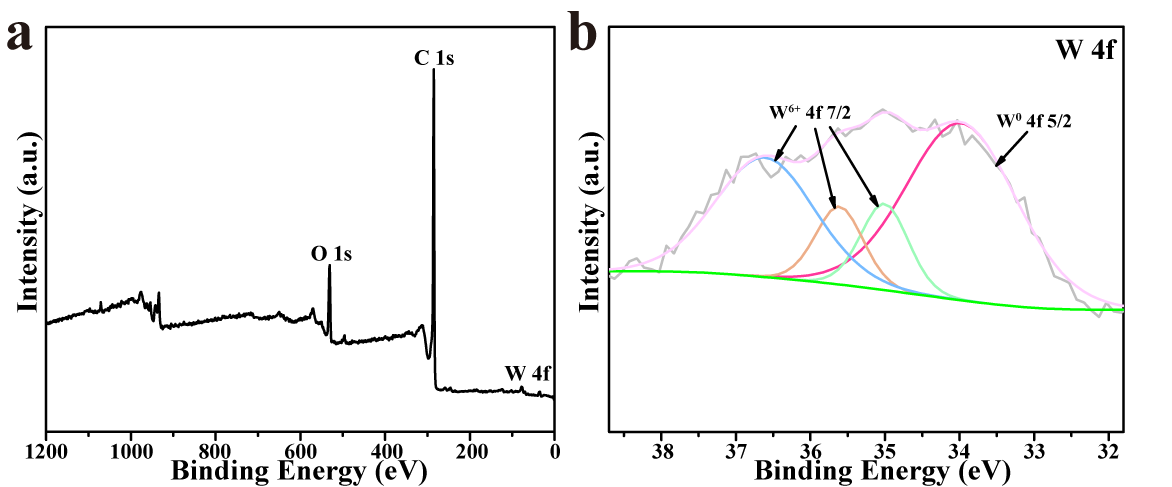


**Figure S11** XPS results of W. a) survey spectrum and b) the high-resolution spectrum of W 4f from the comparative sample W.

**
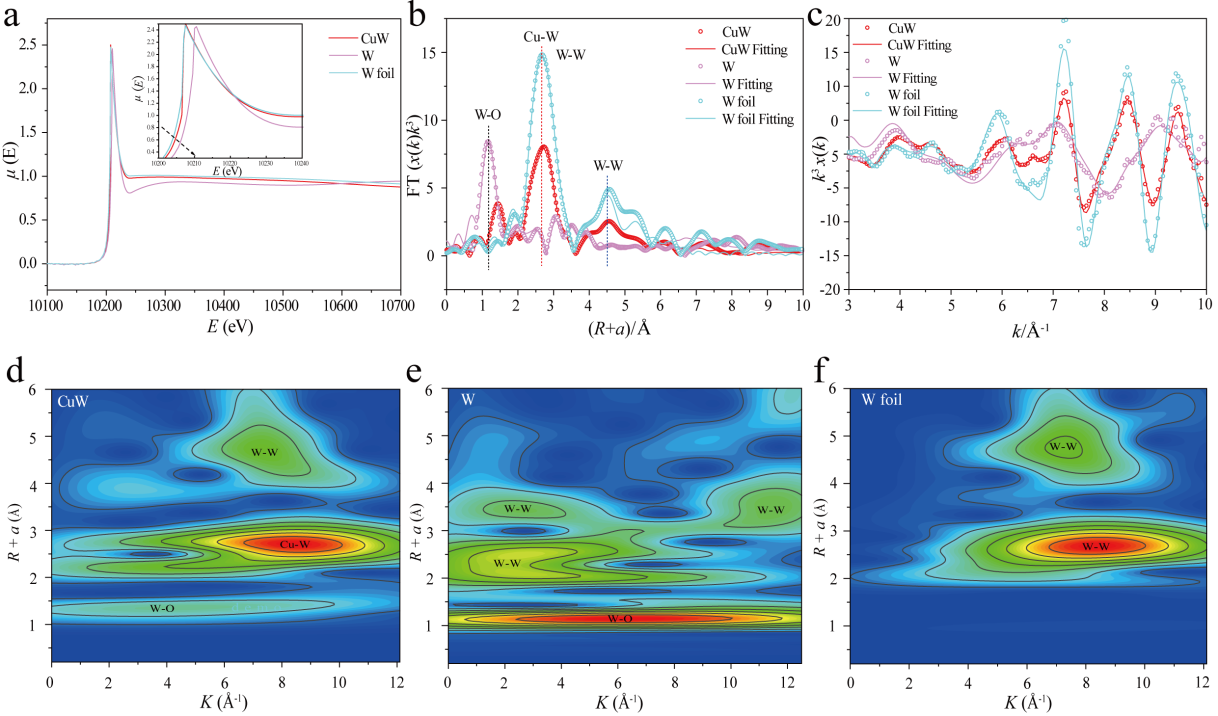
Figure S12** XAFS data of CuW, W, and W foil. a) W *L3*-edge XANES spectra. b) *R*-space extended EXAFS spectra of the materials. c) *K*-space extended EXAFS spectra of the materials. d-f) Wavelet transforms for the *k*^3^–weighted EXAFS signals of CuW (d), W (e), and W foil (f).


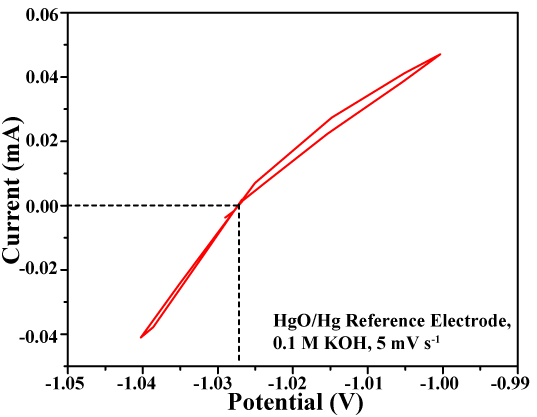


**Figure S13** The RHE calibration of HgO/Hg (filled with 1.0 M KOH) by CV measurement at a scan rate of 5 mV s^–1^ in H_2_-saturated 0.1 M KOH electrolyte at the temperature of 25°C.


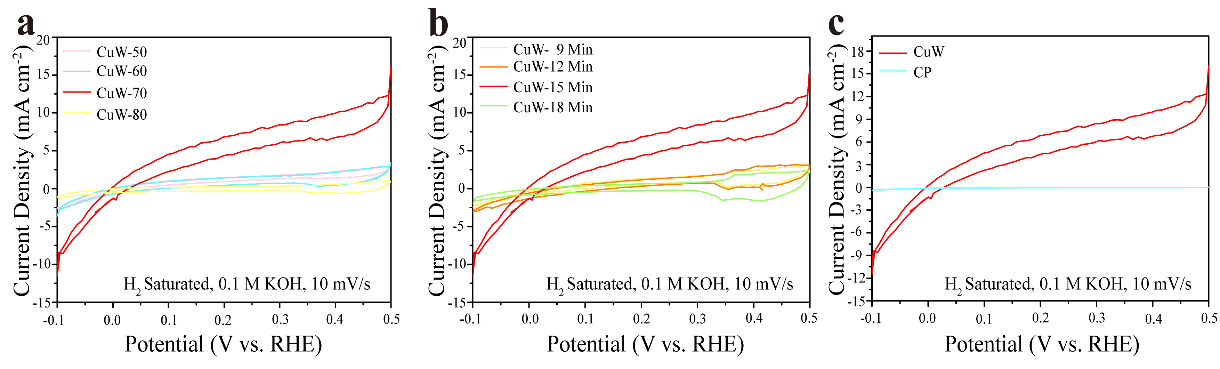
**Figure S14** HOR results of materials. Influences of a) electrodeposition temperature (40ºC, 50ºC, 60ºC, 70ºC); b) electro-deposition time (6 min, 10 min, 14 min, 18 min) on the HOR performances of electrode materials; c) the HOR performance of carbon paper in H_2_-saturated 0.1 M KOH electrolytes by CV measurements.


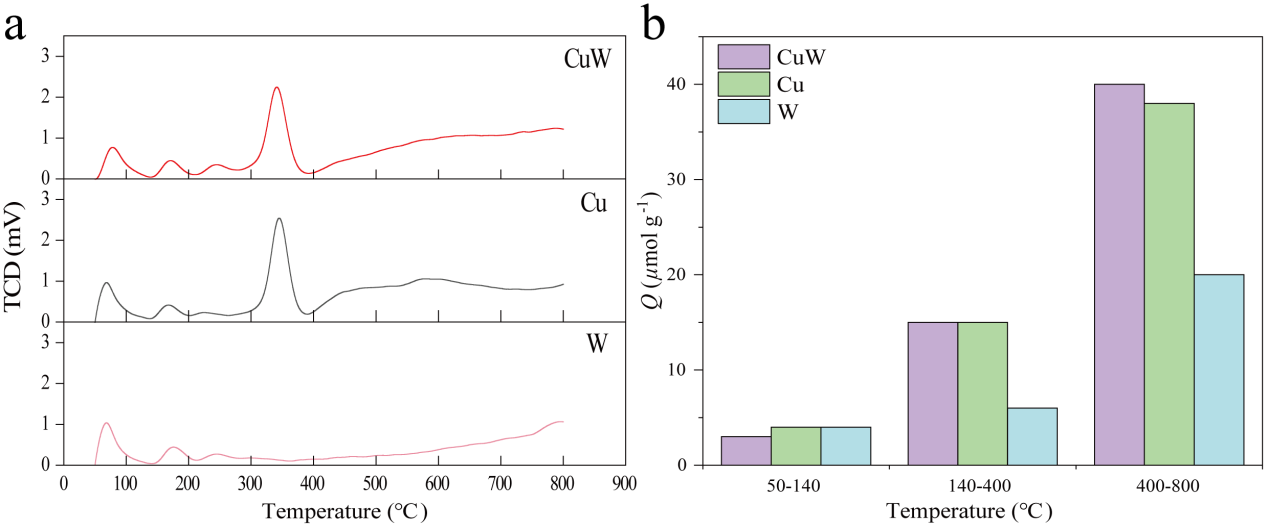


**Figure S15** a) H_2_-TPD profiles of CuW, Cu, and W; b) Bar charts of temperature-dependent hydrogen spillover amounts for CuW, Cu, and W.


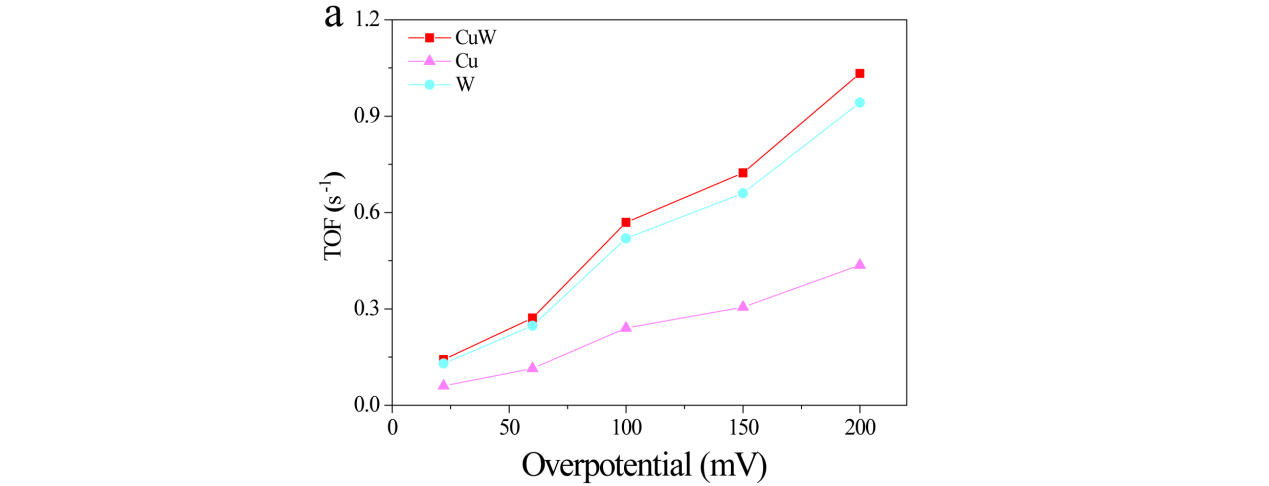


**Figure S16** TOF values of CuW, Cu, W.

The TOF of the materials were calculated with the formula: TOF = *J* × *A* / (4 × *F* × *m*), in which J is the correspond current density at different specific overpotential (A cm^−2^), A is the carbon paper supported catalyst area which is 1 cm^2^ in our study, F is the Faraday constant (96485 mol C^−1^) and m is the number of moles of active sites.


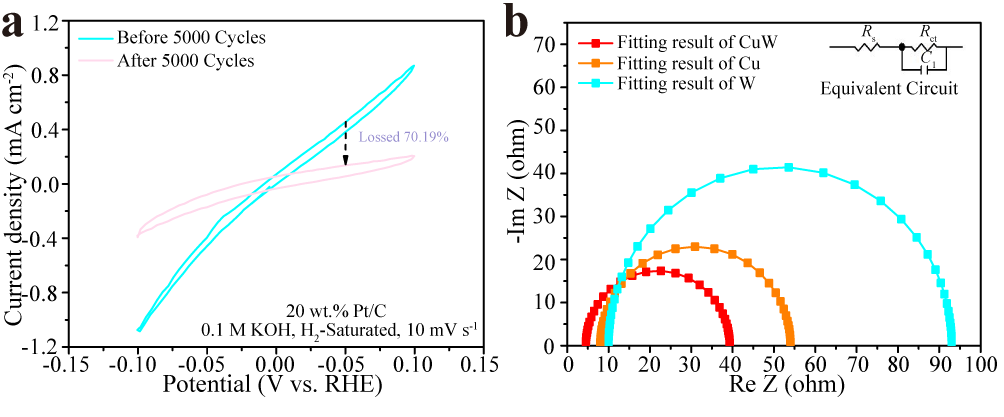


**Figure S17** a) The CV data before and after 5,000 cycles-ADT results of Pt/C in H_2_-saturated 0.1 M KOH electrolyte; b) Fitting results of EIS and the corresponding equivalent circuit diagram measured at open-circuit potentials in 0.1 M KOH electrolytes for CuW, Cu and W.


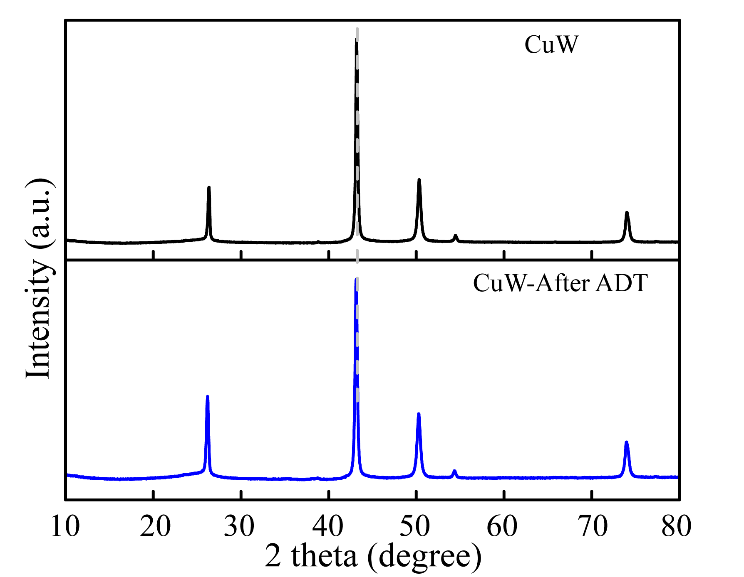


**Figure S18** XRD results of CuW before and after ADT for 1‒20, 000 cycles.

XRD Analysis of CuW (Matching Cu Reference PDF#04-0836)

Peak Matching and Shift Analysis：1. (111) peak, standard 2*θ* (PDF#04-0836): 43.297°, observed 2*θ*: 43.2° (shift: -0.097°), intensity: 1,561,708.5; 2. (200) peak, Standard 2*θ* (PDF#04-0836): 50.43°, observed 2*θ*: 50.40 (shift: -0.033°), intensity: 536,858; 3. (220) peak, standard 2*θ* (PDF#04-0836): 74.13°, observed 2*θ*: 74.10 (shift: -0.030°), intensity: 126,242.

For CuW, the characteristic peaks of Cu (referenced to PDF#04-0836) are as follows:

(111) peak: Standard 2θ ≈ 43.30° (actual 2θ ≈ 43.20, intensity 1,561,709);

(200) peak: Standard 2θ ≈ 50.43° (actual 2θ ≈ 50.40, intensity 536,858);

(220) peak: Standard 2θ ≈ 74.13° (actual 2θ ≈ 74.10, intensity 126,242).

1. Calculating lattice constants and lattice distortion

For cubic systems, the lattice constant *a* = *d*$\sqrt{\text{h}^{\text{2}}\text{+}\text{k}^{\text{2}}\text{+}\text{l}^{\text{2}}}$. For the (111) peak, the standard value for Cu is *a*_Cu_ = 3.615 Å. The measured value here is 2*θ* = 43.0°, so the measured *d*-spacing is: *d*_measured_ = *λ /*2sin*θ* ≈ 2.094  Å, corresponding to a measured lattice constant: *α*_measured_ = 2.094⋅$\sqrt{\text{3}}$≈ 3.625  Å. This indicates slight lattice expansion (due to W solid solution or defects).

1. Determining the full width at half maximum (FWHM, *β*) of the Cu phase in CuW

In calculating the defect density in CuW based on the XRD-measured FWHM (*β*) using the Williamson-Hall (W-H) method or Scherrer equation, the specific steps and calculation results are as follows: the full width at half maximum (FWHM, *β*) results for the (111), (200), and (220) diffraction peaks in the XRD data of CuW are as follows:

(111) diffraction peak at 43.20°, *β* = 0.280°

(200) diffraction peak at 50.40°, *β* = 0.350°

(220) diffraction peak at 74.10°, *β* = 0.450°

According to the Scherrer formula: *D* = *Kλ* /*β*cos*θ*

*D*: Crystallite size (nm), *K*: Scherrer constant (typically 0.89–1.0, here taken as 0.9), *λ*: X-ray wavelength (Cu *Kα*, *λ* = 0.154 nm), *β*: FWHM in radians (1° = π/180 rad), *θ*: Bragg angle (2*θ*/2).

Thus, *D*_111_ ≈ 30.5 nm, *D*_200_ ≈ 25.1 nm, *D*_220_ ≈ 22.1 nm, Average crystallite size: 25.9 nm,

Dislocation density (*ρ*) calculation: *ρ =* 2$\sqrt{\text{3}}$ ${\text{(}\text{ϵ}^{\text{2}}\text{)}}^{\text{1/2}}$/ *Db*, *b*: Burgers vector (for Cu, b ≈ 0.256 nm), ${\text{(}\text{ϵ}^{\text{2}}\text{)}}^{\text{1/2}}$: Microscopic strain (obtained through W-H analysis).

Williamson-Hall (W-H) analysis of grain size and microstrain: *β*cos*θ* = *Kλ /*D+4*ϵ*sin*θ*.

By plotting the linear fit of *β*cos*θ* vs. 4sin*θ*, where the intercept corresponds to *D* and the slope corresponds to the *ϵ*, *ϵ* is approximately 0.001 (tensile strain), and the intercept *Kλ/D* ≈ 0.004, yielding *D* ≈ 34.7 nm.

Calculation of defect density: *ρ=*2$\sqrt{\text{3}}{\text{(}\text{ϵ}^{\text{2}}\text{)}}^{\text{1/2}}$/*Db*=2$\sqrt{\text{3}}$× 0.0012 /(34.7 × 10^-9^ × 0.256 × 10^-9^)$\approx$4.7 × 10^14^ m^-2^.

Cu:

The full width at half maximum (FWHM, *β*) results for the (111), (200), and (220) diffraction peaks in the XRD data of Cu.

(111) diffraction peak, located at 43.3, *β* = 0.24°

(200) diffraction peak, located at 50.4°, *β* = 0.24°

(220) diffraction peak, located at 74.1°, *β* = 0.24°

Based on the Scherrer equation: *D* = *Kλ* /*β* cos*θ*

*D*_111_ = 0.19 × 1.5406 / (0.00419 × cos21.648°) = 356 Å = 35.6 nm

Williamson-Hall (W-H) Analysis for crystallite size and microstrain: *β*cos*θ* = *Kλ /D* + 4*ϵ*sin*θ*. Substituting the relevant data, we obtain: *ϵ* ≈ 0.0005 (Tensile strain).

Intercept: *Kλ /D*$\approx$0.0036, thus, *D*$\approx$ 38.5 nm (Close agreement with the Scherrer formula results).

Defect density calculations: *ρ =* 2$\sqrt{\text{3}}{\text{(}\text{ϵ}^{\text{2}}\text{)}}^{\text{1/2}}$/*Db* = 2$\sqrt{\text{3}}$× 0.0005 /(38.5 × 10^-9^ × 2.56 × 10^-8^)$\approx$1.8 × 10^12^ m^-2^.

CuW after stability measurement:

FWHM of XRD (111), (200), (220) diffraction peaks:

(111) diffraction peak, located at 43.34°，*β*=0.70°

(200) diffraction peak, located at 50.50°，*β*=0.60°

(220) diffraction peak, located at 74.00°，*β*=0.60°

Based on the Scherrer equation: *D* = *Kλ* /*β* cos*θ*

*D*_111_ = 0.19×1.5406 / (0.0122 × cos21.670°) = 258 Å = 25.8 nm

*D*_200_ = 0.19×1.5406 / (0.0105 × cos25.250°) = 308 Å = 30.8 nm

*D*_220_ = 0.19×1.5406 / (0.0105 × cos37.000°) = 118 Å = 34.9 nm

Williamson-Hall (W-H) Analysis for crystallite size and microstrain:

*Β*cos*θ* = *Kλ /D* + 4*ϵ*sin*θ*. Substituting the relevant data, we obtain: *ϵ* ≈ -0.002 (Compressive Strain), *D*$\approx$9.9 nm.

Defect density calculations: *ρ =* 2$\sqrt{\text{3}}{\text{(}\text{ϵ}^{\text{2}}\text{)}}^{\text{1/2}}$/*Db* = 2$\sqrt{\text{3}}$× 0.002 / (9.9 × 10^−9^ × 2.56 × 10^-8^)$\approx$2.7 × 10^13^ m^-2^

**
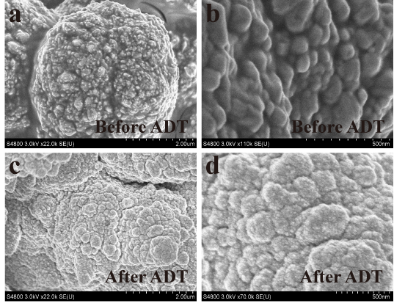
**

**Figure S19** a, c) Low- and b, d) high-resolution SEM images of CuW after 20, 000 cycles of ADT.

**
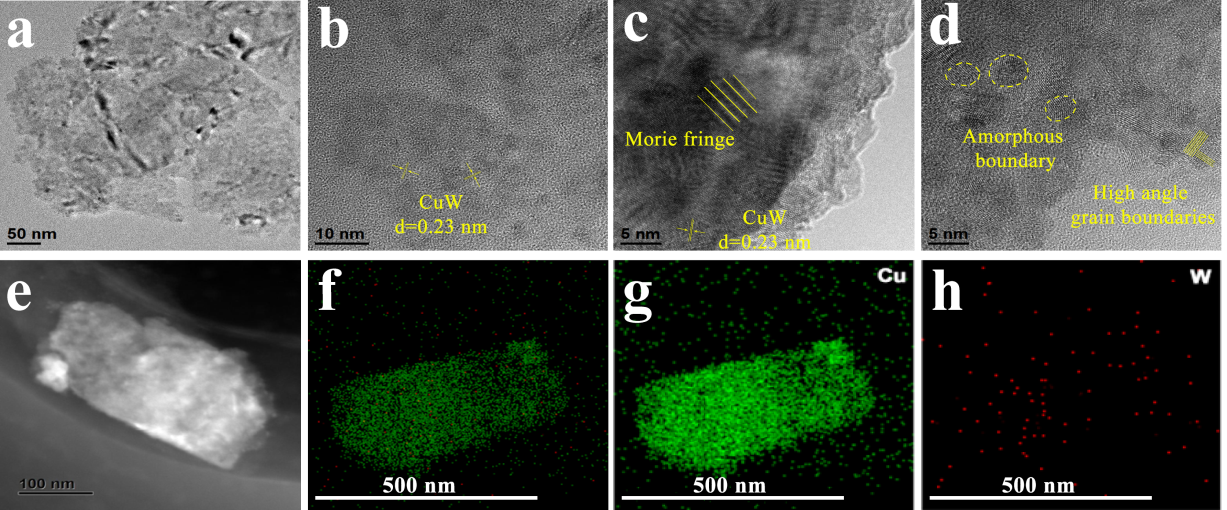
**

**Figure S20** a) Low- and b, c, d) high-magnification images of CuW after 20, 000 cycles of ADT; its corresponding e) STEM-HAADF image and f) EDS elemental mapping images of g) Cu and h) W.

**
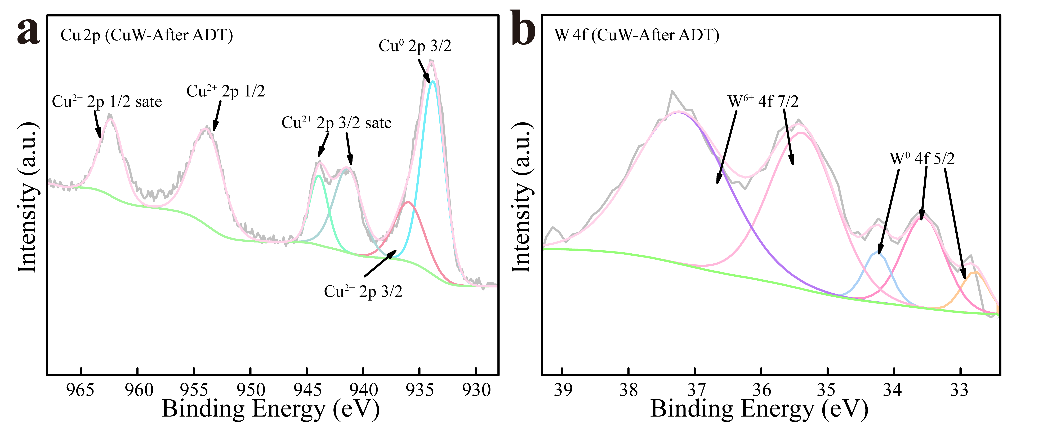
**

**Figure S21** XPS spectra of CuW after 20, 000 cycles of ADT. High-resolution XPS images of a) Cu 2p and b) W 4f from CuW after 20, 000 cycles of ADT.

**
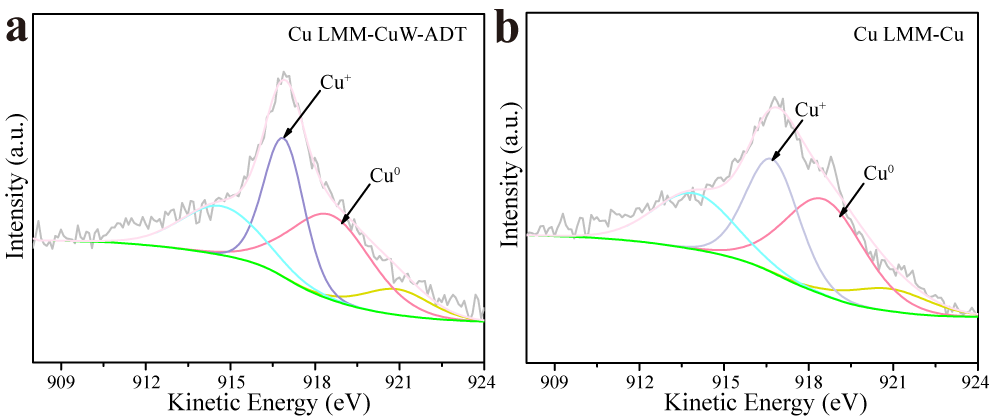
**

**Figure S22** Auger electron spectra of (a) CuW after 20, 000 cycles of ADT and (b) Cu.

**
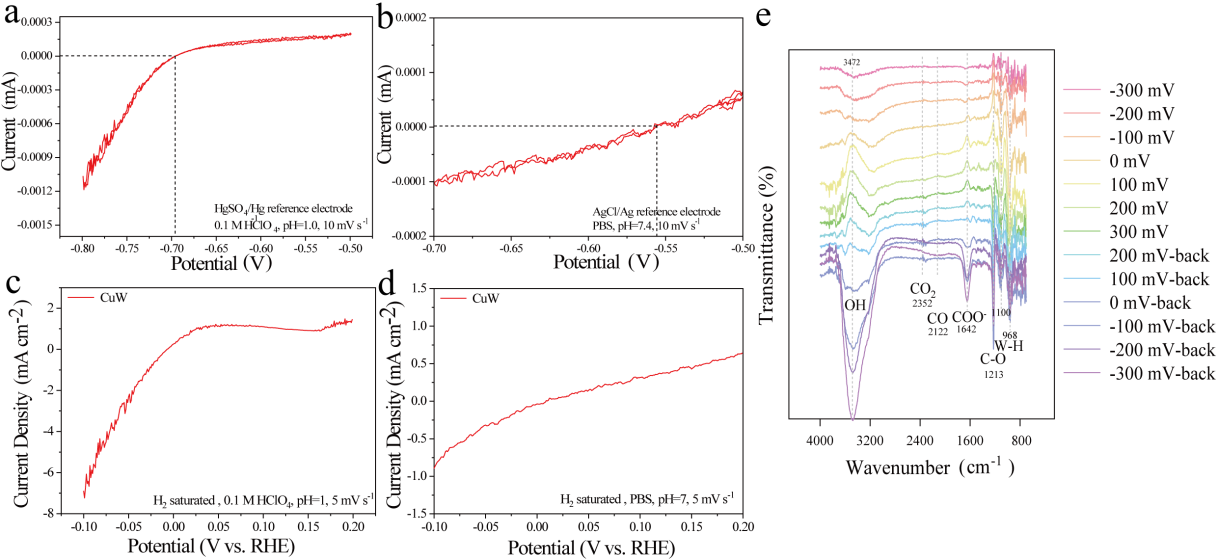
**

**Figure S23** Calibration results of the a) HgSO_4_/Hg, b) AgCl/Ag reference electrodes. CuW HOR performances at c) pH = 1 and d) pH = 7. e) *In-situ* IR spectroscopy performed in the 1, 000 ppm CO/H_2_-saturated 0.1 M KOH electrolyte results of CuW.

**Computational Details**

All the first-principles calculations were performed by using Vienna ab initio simulation package.^1, 2^The ion-electron interactions were described by the projector augmented wave method.^3^ The generalized gradient approximation in the revised-Perdew–Burke–Ernzerhof (RPBE) form and a cut-off energy of 400 eV for plane-wave basis set were adopted.^4^ The convergence criterion for the residual force and energy was set to 0.03 eV Å^-1^ and 10^-5^ eV, respectively, during the structure relaxation.^5, 6^ The minimum energy pathway is determined by using the climbing nudged elastic band method, and the structure of each transition state is optimized until the maximum force on any atom was below 0.05 eV Å^-1^. The Brillouin zones were sampled by a 3 × 3 × 1 *k*-point grid. A vacuum space over 15 Å was employed to avoid the interaction between two periodic units.

The adsorption energy of H_2_ were computed by:

*E*_b_ = *E*_Total_ – *E*_Surface_ – *E*_H2_

where the *E*_Total_, *E*_Surface_ and *E*_H2_ are the energies of the surface with and without the adsorption of H_2_, and the energy of the H_2_ molecule, respectively. Therefore, more negative Δ*E*_b_ represents stronger binding strength.

The adsorption energy of *OH were computed by:

*E*_b_ = *E*_*OH_ + 1/2*E*_H2_ – *E*_Surface_ – *E*_H2O_

where the *E*_*OH_ and *E*_H2O_ are the energies of the surface with the adsorption of *OH, and the energy of the H_2_O molecule, respectively.

Free energy for hydrogen adsorption (Δ*G*_H_)^7, 8^ is computed by:

Δ*G*_H_ = Δ*E*_H_ + Δ*E*_ZPE_ – TΔ*S*_H_

where the Δ*E*_H_, Δ*E*_ZPE_ and Δ*S*_H_ are the adsorption energy of H, zero-point energy difference and the entropy difference between the adsorbed and the gas phase, respectively.

Optimized structures of intermediates on Cu (111), W (110), CuW (111) and CuW-WO_3_ (111) surfaces


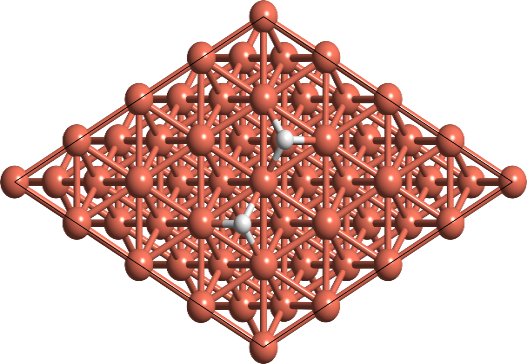

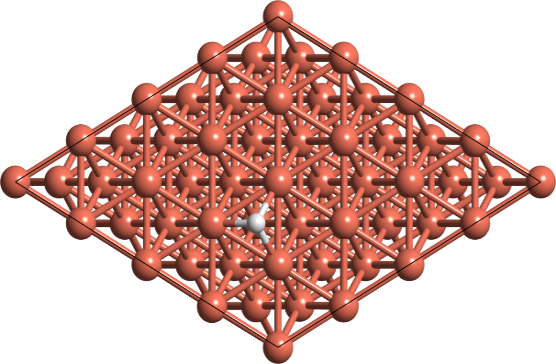


(a) 2*H (b) *H


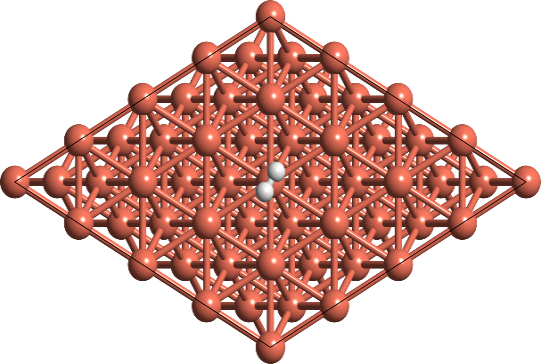

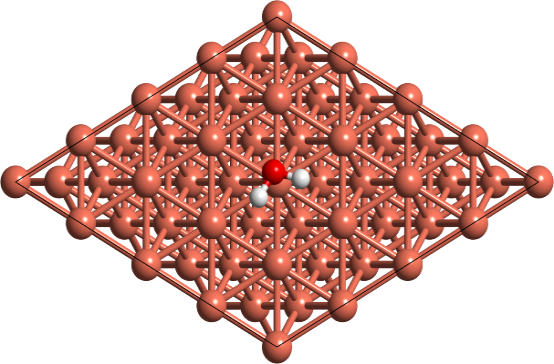


(c) *H_2_ (d) *H_2_O


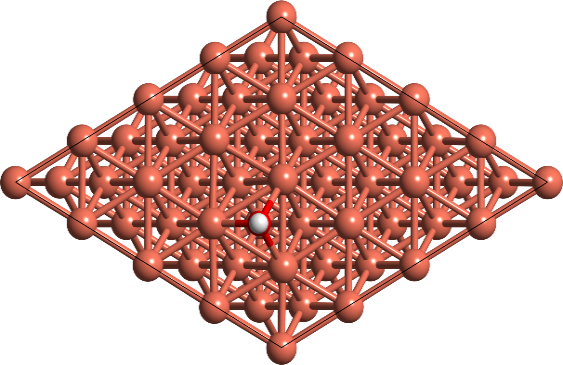

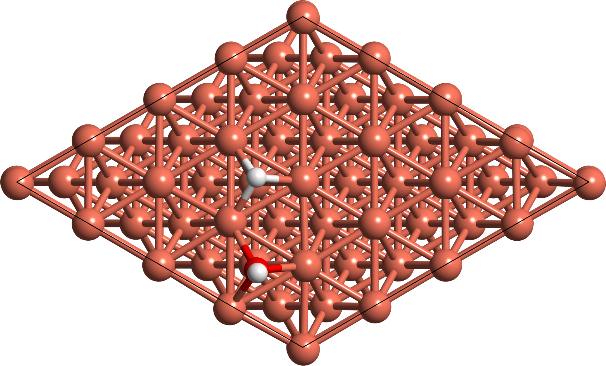


(e) *OH (f) *OH+*H

**Figure S24** Optimized structures of intermediates on Cu (111) surface


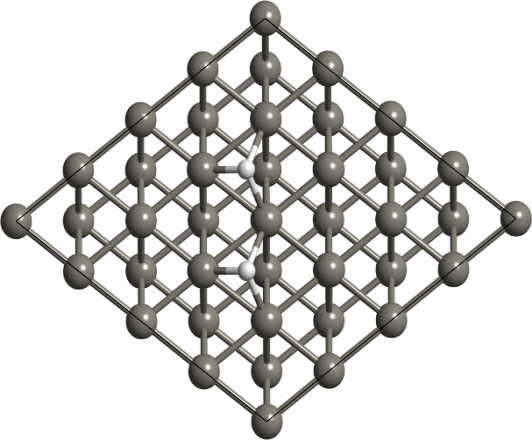

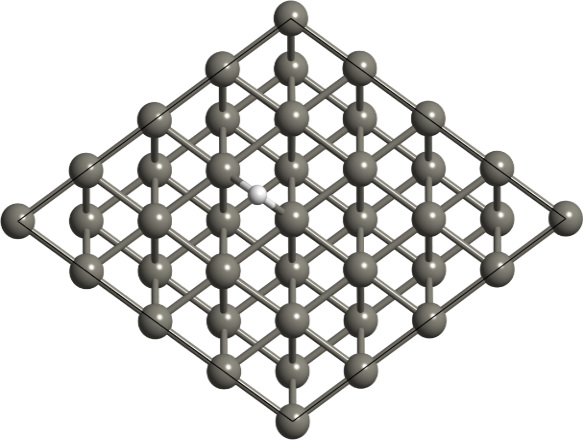


(a) 2*H (b) *H


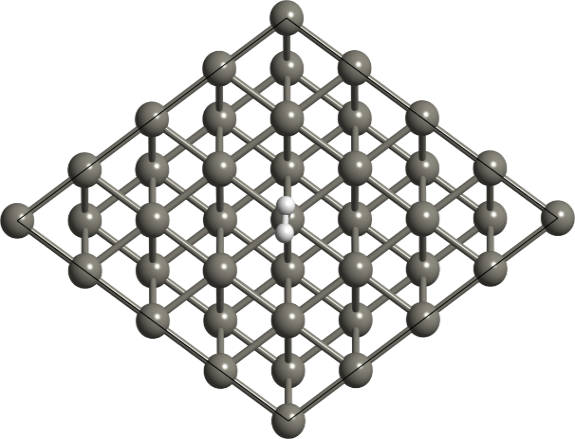

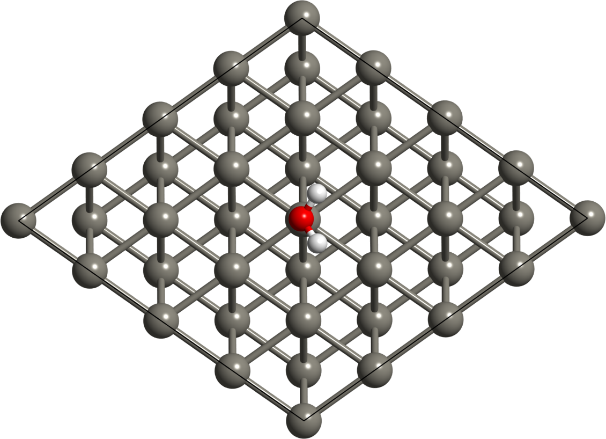


(c) *H_2_ (d) *H_2_O


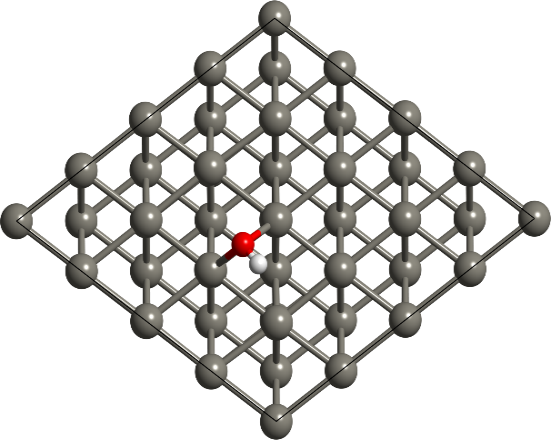

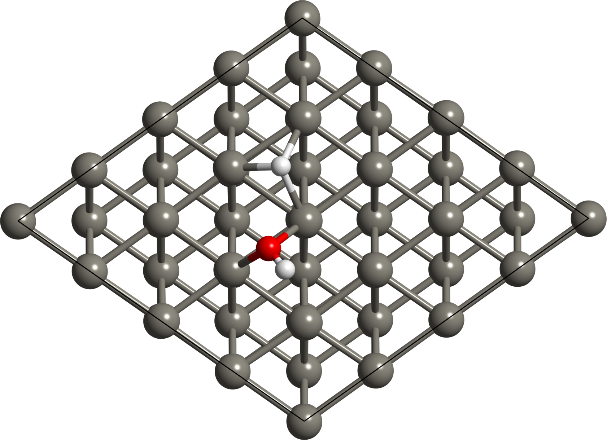


(e) *OH (f) *OH+*H

**Figure S25** Optimized structures of intermediates on W (110) surface


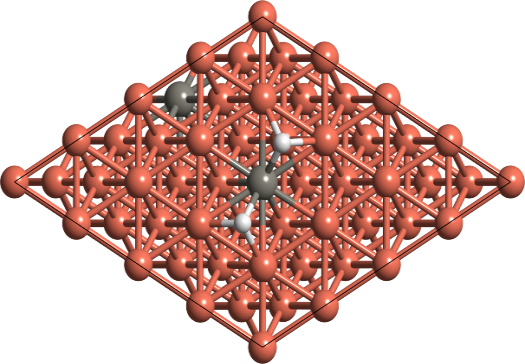

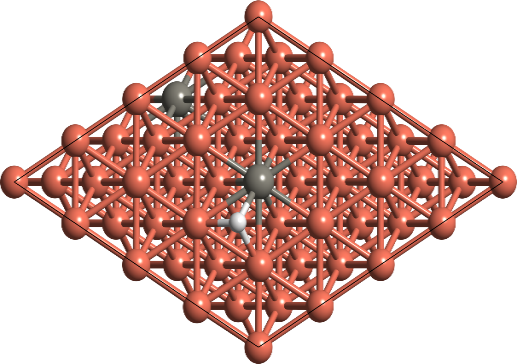


(a) 2*H (b) *H


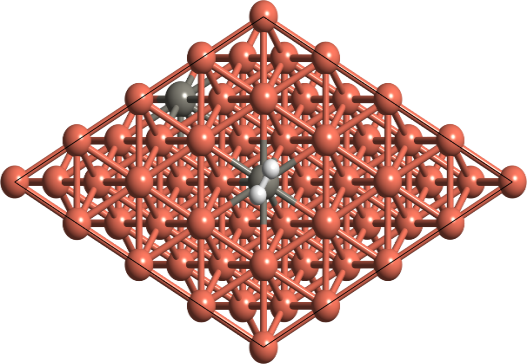

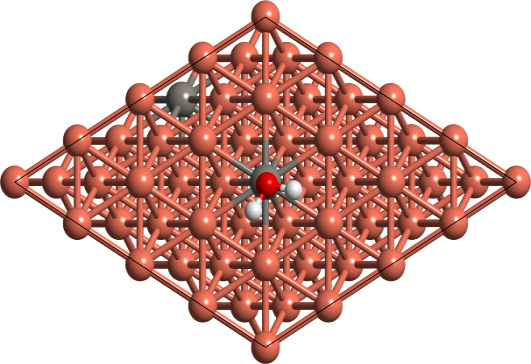


(c) *H_2_ (d) *H_2_O


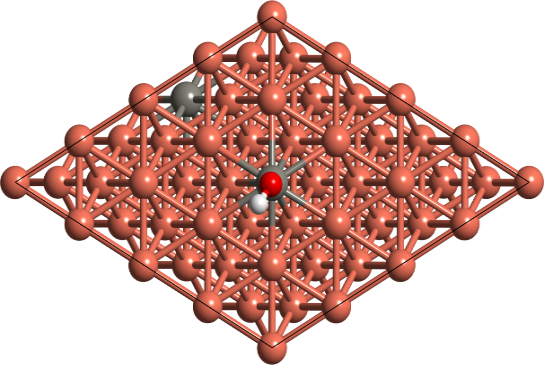

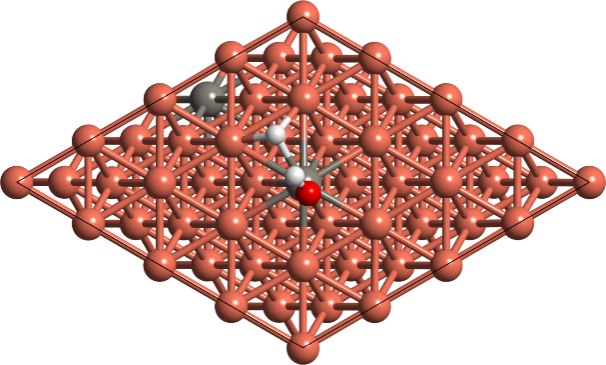


(e) *OH (f) *OH+*H

**Figure S26** Optimized structures of intermediates on CuW (111) surface


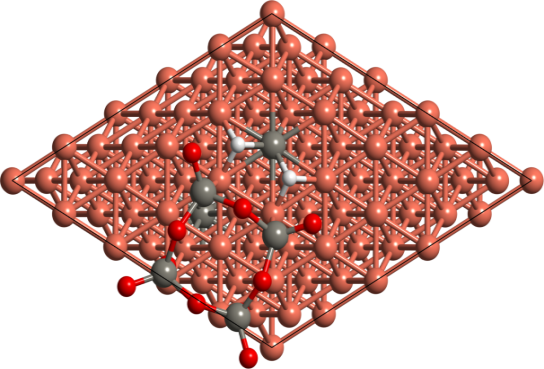

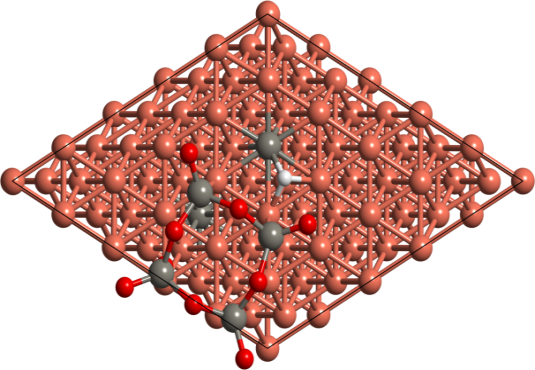


(a) 2*H (b) *H


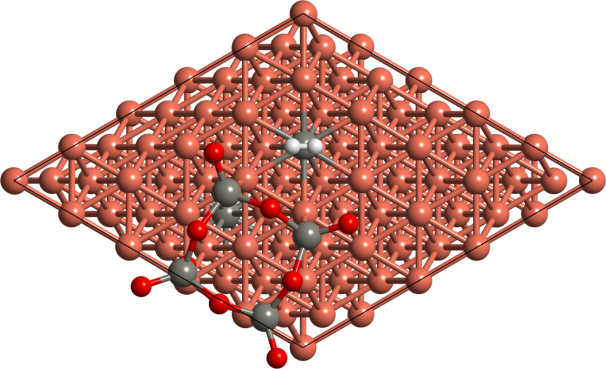

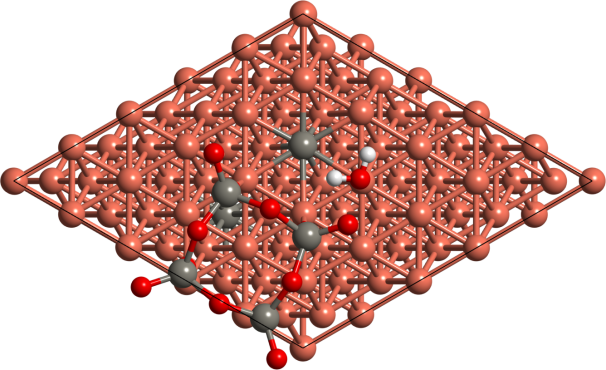


(c) *H_2_ (d) *H_2_O


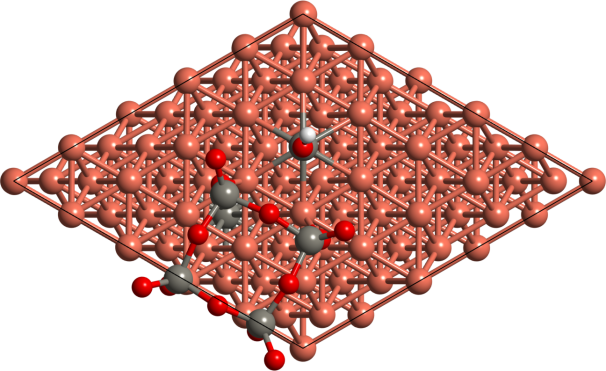

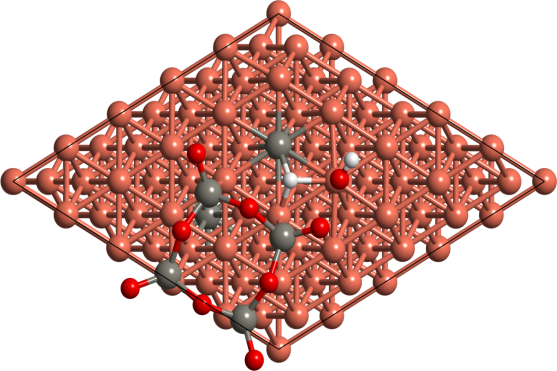


(e) *OH (f) *OH+*H

**Figure S27** Optimized structures of intermediates on CuW-WO_3_ (111) surface

**
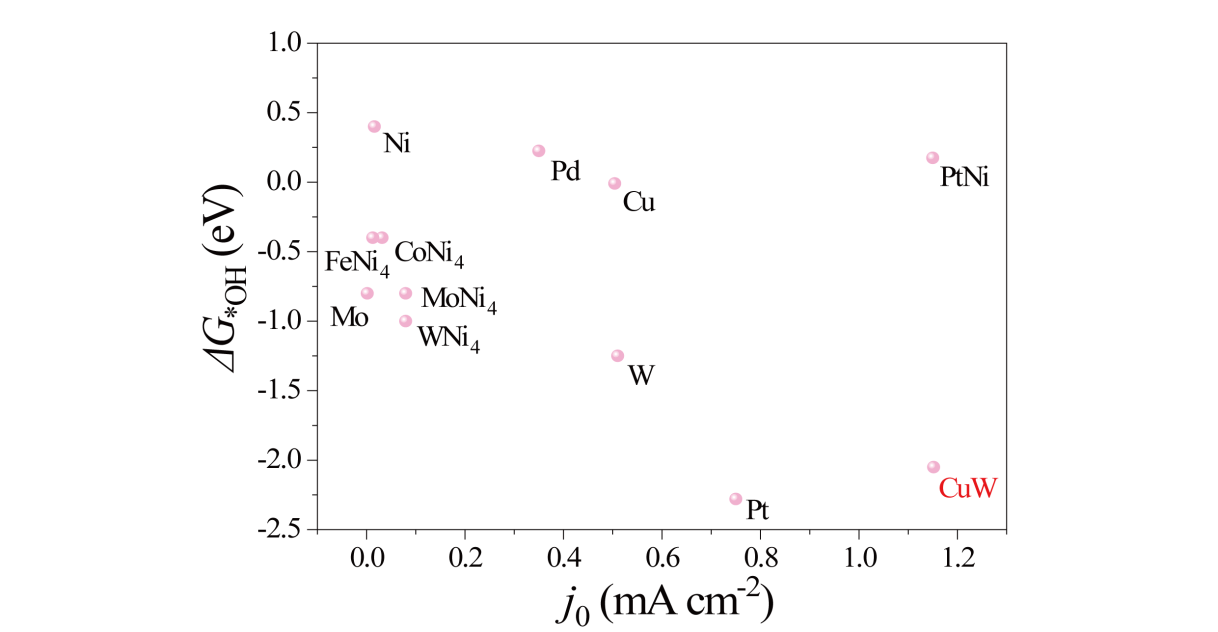
**

**Figure S28** Different catalysts plotted with the calculated *△*G_*OH_ binding energies.


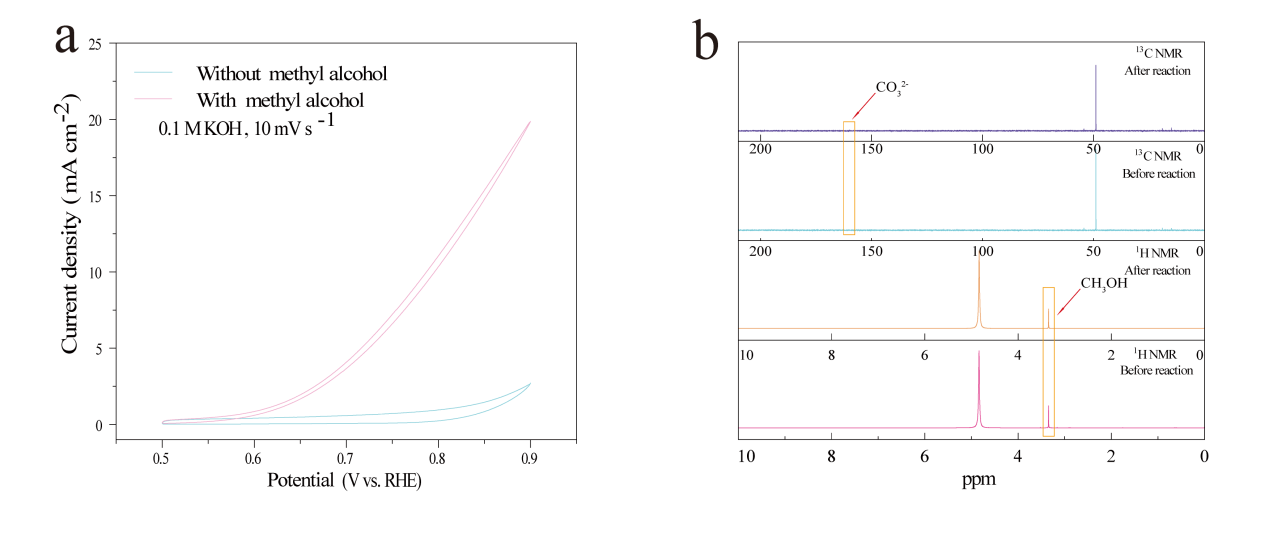


‌**Figure S29**‌ a) Methanol oxidation (MOR) performance tested in 0.1 M KOH with 2.5 mol L^-1^ methanol solution, and b) ^1^H NMR and ^13^C NMR spectra of the electrolyte before and after the reaction.


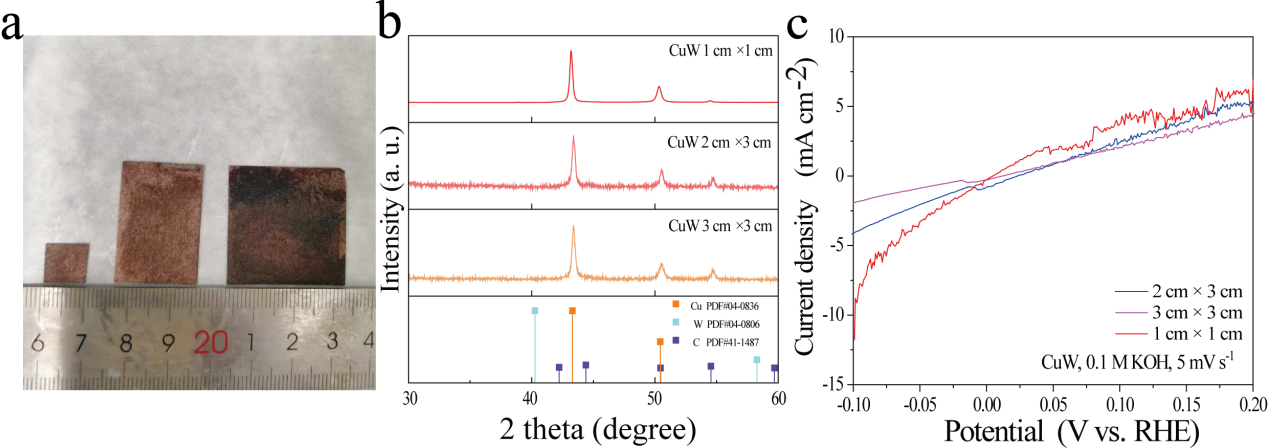


**Figure S30** a) Photographs of CuW catalyst membranes of 1 cm × 1 cm, 2 cm × 3 cm, and 3 cm × 3 cm in sizes. b) XRD patterns of CuW with varying dimensions. c) HOR polarization curves of CuW samples with varying dimensions.

**Table S1** The influences of synthesize temperatures on CuW measured by XRD.

| Materials | (111) | (200) |
| --- | --- | --- |
| CuW (50ºC) | 43.13º | 50.25º |
| CuW (60ºC) | 43.18º | 50.30º |
| CuW (70ºC) | 43.18º | 50.36º |
| CuW (80ºC) | 43.24º | 50.36º |
| Standard Cu | 43.30º | 50.43º |

**Table S2** The influences of synthesize molar ratios on CuW measured by XRD.

| Materials | (111) | (200) |
| --- | --- | --- |
| Cu:W (3:14) | 43.24º | 50.36º |
| Cu:W (6:14) | 43.18º | 50.36º |
| Cu:W (12:14) | 43.24º | 50.36º |
| Cu:W (12:0) | 43.30º | 50.43º |
| Standard Cu | 43.30º | 50.43º |

**Table S3** Molar percentages of chemical bonds in Cu 2p on the surface of Cu.

| Element | Peak position | Chemical bond | Area CPS. eV | Area ratio | Oxidation/Reduction state ratio |
| --- | --- | --- | --- | --- | --- |
| Cu 2p | 932.9 | Cu | 2791 | 4.81% | 56.00%  /44.00% |
|  | 934.2 | Cu | 22738 | 39.19% |  |
|  | 940.4 | Cu-O | 7844 | 13.52% |  |
|  | 943.7 | Cu-O | 6497 | 11.20% |  |
|  | 953.9 | Cu-O | 11735 | 20.25% |  |
|  | 962.4 | Cu-O | 6416 | 11.06% |  |

**Table S4** Molar percentages of chemical bonds in W 4f and Cu 2p on the surface of CuW.

| Element | Peak position | Chemical bond | Area CPS. eV | Area ratio | Oxidation/Reduction state ratio |
| --- | --- | --- | --- | --- | --- |
| W 4f | 33.7 | W | 199.6 | 9.59% | 63.26%  /36.74% |
|  | 34.5 | W | 564.9 | 27.15% |  |
|  | 36.7 | W-O | 1098 | 52.74% |  |
|  | 38.9 | W-O | 218.9 | 10.52% |  |
| Cu 2p | 930.7 | Cu-C | 1051 | 2.23% | 44.34%  /55.66% |
|  | 932.0 | Cu | 5567 | 11.82% |  |
|  | 933.7 | Cu | 15973 | 33.92% |  |
|  | 936.8 | Cu-O | 674.2 | 1.43% |  |
|  | 940.2 | Cu-O | 6497 | 13.80% |  |
|  | 943.2 | Cu-O | 4140 | 8.79% |  |
|  | 950.3 | Cu | 1248 | 2.65% |  |
|  | 951.9 | Cu | 3893 | 8.27% |  |
|  | 953.7 | Cu-O | 4599 | 9.77% |  |
|  | 961.8 | Cu-O | 3443 | 7.31% |  |

**Table S5** Molar percentages of chemical bonds in W 4f on the surface of W.

| Element | Peak position | Chemical bond | Area CPS. eV | Area ratio | Oxidation/Reduction state ratio |
| --- | --- | --- | --- | --- | --- |
| W 4f | 34.0 | W | 662.0 | 49.77% | 50.23%  /49.77% |
|  | 35.0 | W-O | 140.0 | 10.52% |  |
|  | 35.6 | W-O | 126.2 | 9.49% |  |
|  | 36.6 | W-O | 402.0 | 30.22% |  |

**Table S6** Cu-*K E*_0_ values of CuW, Cu, Cu foil

| Sample | CuW | Cu | Cu foil |
| --- | --- | --- | --- |
| *E_0_* value (eV) | 8990 | 8990 | 8990 |

**Table S7** The fitting parameters for the EXAFS data at the Cu *K*-edge the CuW

| Sample | Path | *N* | *S*_0_^2^ | *σ*^2^ (Å^2^) |  | *ΔE_0_* (eV) | | *R* factor |
| --- | --- | --- | --- | --- | --- | --- | --- | --- |
| CuW | Cu-Cu | 9 | 1.158 | 0.009 |  | | 3.808 | 0.0041 |
|  | W | 1 | 7.390 | 0.006 |  | | 15.06 | 0.004 |
|  | Cu-Cu | 14 | 1.368 | 0.015 |  | | -29.23 | 0.007 |

A brief explanation of each parameter:

Coordination number (*N*): The number of atoms that are coordinated or bonded to the central atom，which provides the information about the local atomic environment of the absorber atom.

Amplitude reduction factor (*S_0_*^2^): The attenuation of the photoelectron wave function due to interactions with other atoms in the material.

Mean square relative displacement (*σ*^2^): It quantifies the average squared displacement of atoms from their equilibrium positions and gives a measure of the atomic disorder or vibrations in the material.

Inner potential correction (*ΔE_0_*): It quantifies the effects of the inner atomic potential on the photoelectron wave function.

Goodness of Fit (*R* factor): It quantifies the agreement between the theoretical model and the experimental EXAFS data. A lower R factor indicates a better fit between the data and the model.

The ranges provided for CuW indicate the suitable *k* and *R* ranges for EXAFS data, which are based on the expected structural features and the quality of the experimental data.

Ranges for CuW:

For Cu *K*-edge *k* (Å) between 2.5 and 15.0, *R* (Å) between 1.0 and 4.0.

**Table S8** The fitting parameters for the EXAFS data at the W *L3-*edge the CuW

| Sample | Path | *N* | *S*_0_^2^ | *σ*^2^ (Å^2^) | *ΔE_0_* (eV) | | *R* factor | |
| --- | --- | --- | --- | --- | --- | --- | --- | --- |
| CuW | W-O | 1 | 7.511 | 0.013 | -30.98 | 0.02 | |  |
|  | W-Cu | 21 | 0.112 | -0.013 | 36.12 |  |  |  |
|  | W-Cu | 12 | 0.238 | 0.009 | -7.269 |  |  |  |
|  | W-Cu-Cu | 48 | -2.013 | 0.002 | -13.67 |  |  |  |
|  | W-Cu-Cu | 84 | -0.677 | -0.014 | -29.87 |  |  |  |
|  | W-Cu | 6 | -0.677 | -0.002 | -16.80 |  |  |  |
|  | W-Cu-Cu | 84 | 4.612 | 0 | 6.566 |  |  |  |
|  | W-Cu-Cu | 48 | 31.73 | 0.011 | -8.228 |  |  |  |
|  | W-Cu-Cu | 48 | 1.102 | -0.022 | 17.34 |  |  |  |
|  | W-Cu-Cu | 24 | -7.577 | 0.005 | 17.34 |  |  |  |

**Table S9** The fitting parameters for the EXAFS data at the W *L3-*edge the W

| Sample | Path | *N* | *S*_0_^2^ | *σ*^2^ (Å^2^) |  | *ΔE_0_* (eV) | | *R* factor |
| --- | --- | --- | --- | --- | --- | --- | --- | --- |
| W | W-O | 1 | 11.34 | 0.006 |  | | -92.43 | 0.02 |
|  | W | 8 | 0.464 | 0.008 |  | | -94.07 |  |
|  | W | 6 | 3.850 | 0.036 |  | | 16.68 |  |
|  | W | 12 | 0.004 | -0.031 |  | | -20.43 |  |
|  | W-W | 48 | 0.285 | -0.013 |  | | -19.88 |  |
|  | W-W | 48 | -0.003 | -0.040 |  | | -11.08 |  |

**Table S10** The fitting parameters for the EXAFS data at the W *L3-*edge the W foil

| Sample | Path | *N* | *S*_0_^2^ | *σ*^2^ (Å^2^) |  | *ΔE_0_* (eV) | | *R* factor |
| --- | --- | --- | --- | --- | --- | --- | --- | --- |
| W foil | W | 8 | 1.210 | 0.004 |  | | 7.150 | 0.06 |
|  | W | 6 | 9.834 | 0.026 |  | | 22.99 |  |
|  | W-W | 12 | 0.544 | -0.011 |  | | 0.147 |  |
|  | W-W | 48 | -13.74 | -0.002 |  | | 8.696 |  |
|  | W-W | 48 | -9.793 | -0.005 |  | | 4.482 |  |

The ranges provided for CuW, W and W foil indicate the suitable *k* and *R* ranges for EXAFS data, which are based on the expected structural features and the quality of the experimental data.

Ranges for CuW, W and W foil:

For W *L3*-edge *k* (Å) between 3 and 11 or 12, *R* (Å) between 1.0 and 3.0.

**Table S11** Summary of HOR activities of reported electrocatalysts

| Electrocatalyst | Loading | Electrolyte | *j*_0.05 V vs. RHE_^a^ (mA cm^-2^) | *j*_0,s_^b^ (mA cm^-2^) | Stability of HOR | Ref. |
| --- | --- | --- | --- | --- | --- | --- |
| CuW | 17.9 mg cm^−2^ | 0.10 M KOH | 1.86 | 1.1519 | 20, 000 cycles ADT (-0.1~0.1 V *vs*. RHE) without variation | This work |
| Cu | -^c^ | 0.10 M KOH | 0.69 | 0.5040 | - | This work |
| W | - | 0.10 M KOH | 0.87 | 0.5102 | - | This work |
| Pt/C | 2.5 *μ*g_Pt_ cm^−2^ | 0.10 M KOH | 0.44 | 0.2431 | 5, 000 cycles ADT (-0.1~0.1 V *vs*. RHE) with significant variation | This work |
| WNPC | 3.53 mg cm^−2^ | 0.10 M HClO_4_  0.10 M KOH  0.10 M PBS | 1.03  0.91  0.84 | 0.9342  0.4052  0.5890 | 10000 cycles ADT (-0.1~0.5 V *vs*. RHE) without variation | ^9^ |
| WNC | 3.53 mg cm^−2^ | 0.10 M HClO_4_  0.10 M KOH  0.10 M PBS | 0.81  1.05  0.16 | 0.4078  0.4083  0.4839 | -^c^ | ^9^ |
| Pt/C | 2.5 *μ*g_Pt_ cm^−2^ | 0.10 M HClO_4_  0.10 M KOH  0.10 M PBS | 0.24  0.11  0.16 | 0.1889  0.0553  0.0976 | - | ^9^ |
| Pt (polycrystalline) | - | 0.10 M HClO_4_ | ~0.87 | - | - | ^10^ |
| 5 % Pt/C | 2-20 *μ*g_Pt_ cm^−2^ | 0.10 M KOH | ~2.7 | 0.81 | - | ^11^ |
| PtRu | 14 *μ*g_Pt_ cm^−2^ | 0.10 M KOH | 2.3 | 0.49 | - | ^12^ |
| Ru_0.20_Pt_0.80_/C | 7.09 *μ*g cm^−2^ | 0.10 M KOH | 0.83 mA $\text{cm}_{\text{metal}}^{\text{-2}}$ | 1.42 | - | ^13^ |
| PdNi electrode (Pd:17%) | 0.9 *μ*g_Pd_ cm^−2^ | 0.10 M KOH | 0.30 | 0.35 mA $\text{cm}_{\text{Pd}}^{\text{-2}}$ | - | ^13^ |
| Pd-CN_x_ | 0.043 mg_Pd_ cm^−2^ | 0.10 M H_2_SO_4_ | ~1.5 | 0.84 | - | ^14^ |
| Ir/CeO_2_-C | 13.5 *μ*g _Ir_ cm^−2^ | 0.10 M KOH | ~1.4 | 0.851 | 2000 cycles ADT (0~0.9 V *vs*. RHE) without variation | ^15^ |
| Ir-C | 11.6 *μ*g _Ir_ cm^−2^ | 0.10 M KOH | ~0.7 | 0.356 | 2000 cycles ADT (0~0.9 V *vs*. RHE) without variation | ^15^ |
| (W_0.7_Ir_0.3_)O_y_ | 0.2 mg cm^−2^ | 0.50 M H_2_SO_4_ | ~0.45 | - | - | ^16^ |
| WO_3_-C | 51 mg cm^−2^ | 1.50 M H_2_SO_4_ | ~1.0 | - | - | ^17^ |
| WC | 27 mg cm^−2^ | 1.50 M H_2_SO_4_ | ~3.16 | - | - | ^18^ |
| WC | 1000 mg cm^−2^ | 0.01 M H_2_SO_4_ | ~0.05 | - | - | ^19^ |
| Ni | - | 0.10 M KOH | ~0.10 | - | - | ^20^ |
| Ni/N-CNT | 0.25 *μ*g_metal_ cm^−2^ | 0.10M KOH | ~1.30 | 0.028 mA $\text{cm}_{\text{Ni}}^{\text{-2}}$ | - | ^21^ |
| Ni_0.95_Cu_0.05_/C | 0.25 *μ*g cm^−2^ | 0.10 M NaOH | - | 0.014 | - | ^22^ |
| Ni_3_@(h-BN)_1_/C-700NH_3_ | 0.25 mg_Ni_ cm^−2^ | 0.10 M NaOH | ~1.1 | 0.023 | 10000 cycles ADT (-0.05~0.5 V *vs*. RHE) with some variations | ^23^ |
| CoNiMo(0.12:5.10:1.00) | - | 0.10 M KOH | ~2.00 | - | - | ^20^ |
| WNi | ~0.5 mg cm^−2^ | 0.10 M KOH | ~2.20 | 1.87 mA cm^−2^ | 60 mV *vs*. RHE 20 h with some variations | ^24^ |
| CoWC | 26 *μ*g cm^−1^ | 0.5 M H_2_SO_4_ | ~0.80 | - | - | ^25^ |
| WC | - | 1.0 M H_2_SO4. | ~0.03 mA | - | - | ^26^ |
| WN/rGO | 0.41 mg cm^-2^ | 0.1 M HClO_4_ | ~0.20 | - | - | ^27^ |

^a^ Activity obtained at 0.05 V vs RHE.

^b^ Exchange current density.

^c^ Not mentioned in the article.

**Table S12** Molar percentages of chemical bonds in Cu 2p on the surface of CuW after 20, 000 cycles ADT

| Element | Peak position | Chemical bond | Area CPS. eV | Area ratio | Oxidation/Reduction state ratio |
| --- | --- | --- | --- | --- | --- |
| Cu 2p | 933.8 | Cu | 45886 | 30.62% | 69.38%  /30.62% |
|  | 935.9 | Cu-O | 18185 | 12.14% |  |
|  | 941.4 | Cu-O | 23738 | 15.84% |  |
|  | 943.9 | Cu-O | 12753 | 8.51% |  |
|  | 953.9 | Cu-O | 31205 | 20.83% |  |
|  | 962.3 | Cu-O | 18070 | 12.06% |  |

**Table S13** Molar percentages of chemical bonds in W 4f on the surface of CuW after 20, 000 cycles ADT

| Element | Peak position | Chemical bond | Area CPS. eV | Area ratio | Oxidation/Reduction state ratio |
| --- | --- | --- | --- | --- | --- |
| W 4f | 32.8 | W | 3.95 | 3.64% | 79.31%  /20.69% |
|  | 33.6 | W | 13.7 | 12.58% |  |
|  | 34.3 | W | 4.85 | 4.47% |  |
|  | 35.4 | W-O | 36.6 | 33.71% |  |
|  | 37.2 | W-O | 49.5 | 45.60% |  |

**Table S14** Molar percentages of Cu^+^ and Cu^0^ on the surface of CuW, Cu, CuW -ADT measured by auger electron spectroscopy

| Sample | Chemical valence | Peak position | Area CPS. eV | Area ratio |
| --- | --- | --- | --- | --- |
| CuW | Cu^0^ | 918.6 | 3885 | 39.7% |
|  | Cu^+^ | 916.8 | 3173 | 32.4% |
| Cu | Cu^0^ | 918.6 | 3942 | 38.2% |
|  | Cu^+^ | 916.7 | 3116 | 30.2% |
| CuW-ADT | Cu^0^ | 918.6 | 3543 | 37.8% |
|  | Cu^+^ | 916.9 | 2913 | 31.0% |

**Table S15** EIS data of CuW, Cu and W at open-circuit potentials in 0.1 M KOH electrolyte

| Resistance | CuW | Cu | W |
| --- | --- | --- | --- |
| *R*_s_ | 4.50 Ω | 8.00 Ω | 10.0 Ω |
| *R*_ct_ | 34.8 Ω | 46.0 Ω | 83.0 Ω |
| *C*_1_ | 2 × 10^-2^ F | 2 × 10^-3^ F | 2 × 10^-4^ F |

**Table S16** The calculated intermediate structures energy on CuW (111) surface, unit: eV

|  | CuW (111) | | |
| --- | --- | --- | --- |
|  | *E* | *ΔE* | *Plot E* |
| surface | -233.3 | 0.000 | 0.000 |
| *H_2_ | -240.8 | -0.548 | -0.548 |
| TS | 0.038 | 0.038 | -0.511 |
| 2*H | -241.2 | -0.446 | -0.995 |

**Table S17** The calculated intermediate structures energy on CuW-WO_3_ (111) surface, unit: eV

|  | CuW-WO_3_ (111) | | |
| --- | --- | --- | --- |
|  | *E* | *ΔE* | *Plot E* |
| surface | -508.2 | 0.000 | 0.000 |
| *H_2_ | -515.8 | -0.566 | -0.566 |
| TS | 0.016 | 0.016 | -0.551 |
| 2*H | -516.2 | -0.407 | -0.973 |

**Table S18** The calculated Gibbs free energy of the H adsorption process on CuW-WO_3_ (111) surface, unit: eV

| *G*(*H) | *G*(surface) | *G*(H_2_) | *ΔG*(*H) |
| --- | --- | --- | --- |
| -511.9 | -508.2 | -6.971 | -0.163 |

**Table S19** The calculated adsorption energies of OH^-^

|  | *E*(total) | *E*(surface) | *E*(OH^-^) | *ΔE*(ads) |
| --- | --- | --- | --- | --- |
| Cu (111) | -206.6 | -195.8 | -10.79 | -0.012 |
| W(110) | -782.2 | -770.2 | -10.79 | -1.247 |
| CuW (111) | -222.2 | -209.7 | -10.79 | -1.692 |
| CuW-WO_3_ (111) | -521.1 | -508.2 | -10.79 | -2.046 |

**Table S20** The calculated energy barrier of *H oxidation into H_2_O on CuW-WO_3_ (111) surfaces, unit: eV.

| *State* | *E* | *Plot Energy* |
| --- | --- | --- |
| *H + *OH | -522.1 | 0 |
| TS | 0.572 | 0.572 |
| *H_2_O | -522.2 | -0.167 |

**Table S21** The calculated formation energy of W-doped Cu, unit: eV.

| *E*(doped) | *E*(pure) | *μ*_W_ | *μ*_Cu_ | *ΔE*(formation) |
| --- | --- | --- | --- | --- |
| -126.5 | -119.2 | -4.537 | -0.245 | -2.981 |

**Supplementary References**

(1) Kresse, G.; J., F. Efficient iterative schemes for ab initio total-energy calculations using a plane-wave basis set. *Physical Review B* **1996**, *54* (16), 11169-11186. DOI: 10.1103/PhysRevB.54.11169.

(2) Kresse, G.; Joubert, D. From ultrasoft pseudopotentials to the projector augmented-wave method. *Physical Review B* **1999**, *59* (3), 1758-1775. DOI: 10.1103/PhysRevB.59.1758.

(3) Blöchl, P. E. Projector augmented-wave method. *Physical Review B* **1994**, *50* (24), 17953-17979. DOI: 10.1103/PhysRevB.50.17953.

(4) Hammer, B.; Hansen, L. B.; Norskov, J. K. Improved adsorption energetics within density-functional theory using revised Perdew-Burke-Ernzerhof functionals. *Physical Review B* **1999**, *59*, 7413-7421. DOI: 10.1103/PhysRevB.59.7413.

(5) Henkelman, G.; Uberuaga, B. P.; Jónsson, H. A climbing image nudged elastic band method for finding saddle points and minimum energy paths. *Journal of Chemical Physics* **2000**, *113* (22), 9901-9904. DOI: 10.1063/1.1329672.

(6) Henkelman, G.; Jónsson, H. Improved tangent estimate in the nudged elastic band method for finding minimum energy paths and saddle points. *Journal of Chemical Physics* **2000**, *113* (22), 9978-9985. DOI: 10.1063/1.1323224.

(7) Nørskov, J. K.; Bligaard, T.; Logadottir, A.; Kitchin, J. R.; Chen, J. G.; Pandelov, S.; Stimming, U. Trends in the Exchange Current for Hydrogen Evolution. *Journal of the Electrochemical Society* **2005**, *152* (3), J23-J26. DOI: 10.1002/chin.200524023.

(8) Greeley, J.; Jaramillo, T. F.; Bonde, J.; Chorkendorff, I. B.; Norskov, J. K. Computational high-throughput screening of electrocatalytic materials for hydrogen evolution. *Nature Materials* **2006**, *5* (11), 909-913. DOI: 10.1038/nmat1752.

(9) Xiong, B.; Zhao, W.; Chen, L.; Shi, J. One-Step Synthesis of W_2_C@N,P-C Nanocatalysts for Efficient Hydrogen Electrooxidation across the Whole pH Range. *Advanced Functional Materials* **2019**, *29* (31), 1902505-1902513. DOI: 10.1002/adfm.201902505.

(10) Chung, H. T.; Martinez, U.; Matanovic, I.; Kim, Y. S. Cation-Hydroxide-Water Coadsorption Inhibits the Alkaline Hydrogen Oxidation Reaction. *Journal of Physical Chemistry Letters* **2016**, *7* (22), 4464-4469. DOI: 10.1021/acs.jpclett.6b02025.

(11) Zheng, J.; Sheng, W.; Zhuang, Z.; Xu, B.; Yan, Y. Universal dependence of hydrogen oxidation and evolution reaction activity of platinum-group metals on pH and hydrogen binding energy. *Science Advances* **2016**, *2*, 1501602-1501609. DOI: 10.1126/sciadv.1501602.

(12) Scofield, M. E.; Zhou, Y.; Yue, S.; Wang, L.; Su, D.; Tong, X.; Vukmirovic, M. B.; Adzic, R. R.; Wong, S. S. Role of Chemical Composition in the Enhanced Catalytic Activity of Pt-Based Alloyed Ultrathin Nanowires for the Hydrogen Oxidation Reaction under Alkaline Conditions. *ACS Catalysis* **2016**, *6*, 3895-3908. DOI: 10.1021/acscatal.6b00350.

(13) Bakos, I.; Paszternák, A.; Zitoun, D. Pd/Ni Synergestic Activity for Hydrogen Oxidation Reaction in Alkaline Conditions. *Electrochimica Acta* **2015**, *176*, 1074-1082. DOI: 10.1016/j.electacta.2015.07.109.

(14) Bhowmik, T.; Kundu, M. K.; Barman, S. Palladium Nanoparticle-Graphitic Carbon Nitride Porous Synergistic Catalyst for Hydrogen Evolution/Oxidation Reactions over a Broad Range of pH and Correlation of Its Catalytic Activity with Measured Hydrogen Binding Energy. *ACS Catalysis* **2016**, *6*, 1929-1941. DOI: 10.1021/acscatal.5b02485.

(15) Qin, B.; Yu, H.; Chi, J.; Jia, J.; Gao, X.; Yao, D.; Yi, B.; Shao, Z. A novel Ir/CeO_2_–C nanoparticle electrocatalyst for the hydrogen oxidation reaction of alkaline anion exchange membrane fuel cells. *RSC Advances* **2017**, *7* (50), 31574-31581. DOI: 10.1039/c7ra03675b.

(16) Patel, P. P.; Jampani, P. H.; Datta, M. K.; Velikokhatnyi, O. I.; Hong, D.; Poston, J. A.; Manivannan, A.; Kumta, P. N. WO_3_ based solid solution oxide-promising proton exchange membrane fuel cell anode electrocatalyst. *Journal of Materials Chemistry A* **2015**, *3*, 18296-18309. DOI: 10.1039/c5ta03792a.

(17) Rees, E. J.; Essaki, K.; Brady, C. D. A.; Burstein, G. T. Hydrogen electrocatalysts from microwave-synthesised nanoparticulate carbides. *Journal of Power Sources* **2009**, *188* (1), 75-81. DOI: 10.1016/j.jpowsour.2008.11.074.

(18) Essaki, K.; Rees, E. J.; Burstein, G. T.; Haslam, G. E. Synthesis and Characterisation of Nanoparticulate WC Electrocatalysts. *ECS Transactions* **2009**, *25* (1), 141-153. DOI: 10.1149/1.3210566.

(19) Bodoardo, S.; Maja, M.; Penazzi, N.; Henn, F. E. G. Oxidation of hydrogen on WC at low temperature. *Electrochimica Acta* **1997**, *42* (17), 2603-2609. DOI: 10.1016/S0013-4686(96)00434-3.

(20) Sheng, W.; Bivens, A. P.; Myint, M.; Zhuang, Z.; Forest, R. V.; Fang, Q.; Chen, J. G.; Yan, Y. Non-precious metal electrocatalysts with high activity for hydrogen oxidation reaction in alkaline electrolytes. *Energy and Environmental Science* **2014**, *7* (5), 1719-1724. DOI: 10.1039/c3ee43899f.

(21) Zhuang, Z.; Giles, S. A.; Zheng, J.; Jenness, G. R.; Caratzoulas, S.; Vlachos, D. G.; Yan, Y. Nickel supported on nitrogen-doped carbon nanotubes as hydrogen oxidation reaction catalyst in alkaline electrolyte. *Nature Communications* **2016**, *7*, 10141-10148. DOI: 10.1038/ncomms10141.

(22) Cherstiouk, O. V.; Simonov, P. A.; Oshchepkov, A. G.; Zaikovskii, V. I.; Kardash, T. Y.; Bonnefont, A.; Parmon, V. N.; Savinova, E. R. Electrocatalysis of the hydrogen oxidation reaction on carbon-supported bimetallic NiCu particles prepared by an improved wet chemical synthesis. *Journal of Electroanalytical Chemistry* **2016**, *783*, 146-151. DOI: 10.1016/j.jelechem.2016.11.031.

(23) Gao, L.; Wang, Y.; Li, H.; Li, Q.; Ta, N.; Zhuang, L.; Fu, Q.; Bao, X. A nickel nanocatalyst within a *h*-BN shell for enhanced hydrogen oxidation reactions. *Chemical Science* **2017**, *8*, 5728–5734. DOI: 10.1039/c7sc01615h.

(24) Duan, Y.; Yu, Z.-Y.; Yang, L.; Zheng, L.-R.; Zhang, C.-T.; Yang, X.-T.; Gao, F.-Y.; Zhang, X.-L.; Yu, X.; Liu, R.; et al. Bimetallic nickel-molybdenum/tungsten nanoalloys for high-efficiency hydrogen oxidation catalysis in alkaline electrolytes. *Nature Communications* **2020**, *11* (1), 4789-4798. DOI: 10.1038/s41467-020-18585-4.

(25) Izhar, S.; Yoshida, M.; Nagai, M. Characterization and performances of cobalt–tungsten and molybdenum–tungsten carbides as anode catalyst for PEFC. *Electrochimica Acta* **2009**, *54* (4), 1255-1262. DOI: 10.1016/j.electacta.2008.08.049.

(26) Hara, Y.; Minami, N.; Itagaki, H. Synthesis and characterization of high-surface area tungsten carbides and application to electrocatalytic hydrogen oxidation. *Applied Catalysis A: General* **2007**, *323*, 86-93. DOI: 10.1016/j.apcata.2007.02.011.

(27) Cai, B.; Shen, D.; Xie, Y.; Yan, H.; Wang, Y.; Chen, X.; Wang, L.; Fu, H. Unlocking Superior Hydrogen Oxidation and CO Poisoning Resistance on Pt Enabled by Tungsten Nitride-Mediated Electronic Modulation. *Journal of the American Chemical Society* **2024**, *146* (48), 33193-33203. DOI: 10.1021/jacs.4c12720.
